# Acupuncture treatment of vascular cognitive impairment through peripheral nerve stimulation pathway: a scoping review

**DOI:** 10.3389/fnagi.2025.1515327

**Published:** 2025-04-28

**Authors:** Xinming Yang, Bo Li, Linna Wu, Ying Cui

**Affiliations:** ^1^First Teaching Hospital of Tianjin University of Traditional Chinese Medicine, Tianjin, China; ^2^National Clinical Research Center for Chinese Medicine Acupuncture and Moxibustion, Tianjin, China; ^3^Qinghai Provincial Hospital of Traditional Chinese Medicine, Xining, China; ^4^The First Clinical Medical School, Yunnan University of Chinese Medicine, Kunming, China

**Keywords:** vascular cognitive impairment, peripheral nerve stimulation, central nervous system modulation, acupuncture, electroacupuncture

## Abstract

**Objective:**

This study aims to explore the central effects of acupuncture on vascular cognitive impairment (VCI) through peripheral nerve stimulation.

**Methods:**

This scoping review followed the methodological framework proposed by Arksey and O’Malley and the PRISMA-ScR guidelines. A comprehensive search of databases, including PubMed, Web of Science, MEDLINE, and Embase, was conducted, including 79 studies on acupuncture interventions for VCI. Acupoints and their underlying anatomical structures related to peripheral nerves were summarized, and the potential pathways of acupuncture effects via different peripheral nerves were explored.

**Results:**

The results showed that acupuncture, by stimulating specific acupoints on the head, face, torso, and limbs, significantly affects peripheral nerve networks, including the cervical, lumbar, and sacral plexuses, thoracic nerves, vagus nerve, trigeminal nerve and its branches. The nerve stimulation effects of acupuncture can enhance the regulation of cerebral blood flow, modulate neuroimmune responses, improve brain function, and promote neuroplasticity through multiple central nervous system pathways, ultimately improving cognitive function and treating VCI.

**Conclusion:**

Acupuncture is a treatment modality that influences the central nervous system through peripheral nerve stimulation to treat VCI. A deeper understanding of the central effects induced by acupuncture-triggered neural reflexes can contribute to the improvement of existing therapies and help elucidate the scientific principles underlying acupuncture’s therapeutic effects.

## 1 Introduction

Vascular Cognitive Impairment (VCI) primarily arises from cerebrovascular diseases (van der Flier et al., 2018; Corriveau et al., 2016), manifesting as mild cognitive impairments (MCI) or a combination of ischemic or hemorrhagic cerebral vascular factors. It can also co-occur with vascular dementia (VD) linked to Alzheimer’s disease (AD) (Zhang et al., 2019). Studies predict that around 2050, the global number of individuals affected by VCI will reach 150 million, incurring substantial financial costs (Alzheimers Disease International, 2018). The ongoing global aging population has exacerbated the occurrence of vascular risk factors and intensified the prevalence among the expanding elderly population (Fitzpatrick et al., 2004). In the future, approximately one-third of individuals aged 65 and above may experience a stroke or dementia (Wolf, 2012). Factors such as cerebral vascular narrowing, reduced blood flow, microbleeds, impaired neural circuits, and brain structural damage may contribute to the onset of VCI. Hypoxia, increased blood–brain barrier (BBB) permeability, endothelial dysfunction, systemic inflammation, and the inflammation-associated aging clock (iAge) represent other potential mechanisms (Zlokovic et al., 2020). Multiple infarctions can cause neurodegenerative changes, thus impacting cognitive function (Biesbroek et al., 2013). Risk factors for VCI encompass hypertension (Reitz et al., 2007), obesity (Pedditzi et al., 2016), hyperglycemia (Saczynski et al., 2008), hyperlipidemia (van de Rest et al., 2008; Dangour et al., 2010; Stough et al., 2012), elevated homocysteine (Nalder et al., 2021), and smoking. Symptomatic treatment for VCI often involves using acetylcholinesterase inhibitors and NMDA receptor antagonists to improve cognition. However, drugs like donepezil and galantamine show limited clinical efficacy, providing only mild enhancements in cognitive function, often with no statistically significant difference compared to a placebo (Battle et al., 2021). Hence, continued exploration of pharmaceuticals and other clinical interventions remains necessary.

Neural or peripheral nerve stimulation is an adjunctive alternative therapy for treating cognitive impairments or cerebrovascular diseases, aiming to regulate the central nervous system through peripheral stimulation. For example, ear and Vagus nerve stimulation (VNS) have been repeatedly reported to enhance and improve cognitive function (Wang et al., 2022; Choi et al., 2022). Damage to the autonomic nervous system leads to dysregulation of neural control, resulting in reduced cerebral blood flow (CBF), often associated with a range of nonspecific neurological symptoms (Ratan and Schiff, 2018). The activation of relevant brain areas after brain injury plays an enhanced role in neuroplasticity. Transcranial direct current stimulation affects synaptic plasticity, brain network connectivity, and CBF regulation (Bikson et al., 2016). The underlying principle is to induce changes in transmembrane potentials of neurons through stimulation, thereby affecting neuronal excitatory and inhibitory responses (Nitsche et al., 2012). It can also activate activity in relevant brain areas, aiding in alleviating central sensitization (Esposito et al., 2019). Simultaneously, it modulates vascular constriction and dilation through the vascular neurocoupling mechanism (Sdrulla et al., 2018). Multiple pathways demonstrate the close connection between the peripheral and central nervous systems. The intrinsic mechanisms activated through neural stimulation hold significant therapeutic implications for VCI.

Acupuncture is the most widely used traditional medical complementary and alternative therapy. According to the World Health Organization’s 2019 report, acupuncture is utilized in 113 out of 120 countries surveyed (Zhang et al., 2019). Countries like China, the United States, the United Kingdom, Australia, Japan, South Korea, and Malaysia have established clinical practice guidelines for acupuncture (Wu et al., 2021; Yang et al., 2017). Several high-quality clinical randomized controlled trials have demonstrated the effectiveness of acupuncture in managing various diseases such as obesity, facial paralysis, different types of pain, ischemic stroke, irritable bowel syndrome, depression, and hypertension (Wen et al., 2021; Zhong et al., 2022). With the advancement of modern science and technology, scientists have been continually exploring the physiological mechanisms through which acupuncture operates. Acupuncture therapy, by stimulating acupoints, mobilizes the body’s intrinsic systems and self-regulatory potential, playing a role in disease prevention and treatment. Acupoints serve as the initial response sites to acupuncture stimulation and are the starting point for acupuncture effectiveness (Zhou and Benharash, 2014). In the 1970s, domestically and internationally researchers began investigating the anatomical structure of acupoints. Currently, studies have revealed (Zhou and Benharash, 2014; Cho et al., 2022) that in the trunk region, there are four nerve plexuses of the spinal nerves that reach the skin of the neck, back, waist, abdomen, and sacrum. The subcutaneous area of acupoints corresponds to the same or nearby spinal segments and nerve branches of visceral organs. Subcutaneous tissues in the upper limb acupoint areas are mostly related to the radial nerve, median nerve, and ulnar nerve. They are commonly used to treat diseases of the head, face, chest, and segmental nerve distribution areas. In the lower limb acupoint areas, subcutaneous nerve tissues are mostly related to the sciatic nerve and femoral nerve and their branches (such as the peroneal nerve and tibial nerve) and are commonly used to treat diseases of the lower limbs, abdomen, or pelvic area. Studies have shown (Gong et al., 2020; Torres-Rosas et al., 2014) that acupuncture, as a form of mechanical stimulation, induces biophysical changes in the subcutaneous tissues of the acupoint area. This stimulation excites local nerve receptors and mechanosensitive ion channels (MSCs) in nerve cells and nerve terminals of the acupoint area, leading to their activation and release of relevant chemicals that act on corresponding receptors in nerve terminals. This process transforms mechanical force signals into electrochemical signals, thus exerting the effects of acupuncture. Moreover, acupuncture can also activate MSCs on non-neuronal cells like macrophages, granulocytes, mast cells, and T cells in the acupoint area (Li et al., 2021; Wu et al., 2014; Huang et al., 2018), triggering the release of chemical substances that act on surrounding nerve cells or receptors in nerve terminals. This activation initiates neuroimmune regulation in the acupoint area, converting the physical signal of acupuncture into a biochemical signal and generating subsequent biological effects. This article mainly explores related Neurostimulation therapy of VCI, attempting to summarize the central effects of acupuncture on peripheral nerve stimulation, reveal the mechanism of acupuncture treatment for VCI, and the theory of acupuncture’s neural effects.

## 2 Methods

This scoping review followed the methodological framework proposed by Arksey and O’Malley (2005), which includes identifying the research question, searching for relevant studies, selecting studies, charting the data, and summarizing, synthesizing, and reporting the results based on the PRISMA-ScR (Tricco et al., 2018) (Preferred Reporting Items for Systematic Reviews and Meta-Analyses Extension for Scoping Reviews) guidelines.

### 2.1 Research question of this review

The main focus of this review is to summarize the peripheral nerves involved in acupoint stimulation based on studies related to acupuncture interventions for VCI. The aim is to explore the potential peripheral nerve pathways that may be involved in acupuncture treatment for VCI.

### 2.2 Search strategy

We searched English-language databases, including PubMed, Web of Science, MEDLINE, and Embase. The search terms included subject headings and related terms such as “acupuncture,” “electroacupuncture,” and keywords like “vascular cognitive impairment.” The two sets of keywords were connected using the logical operator “AND.” The search covered the period from the inception of each database up to March 27, 2024, and included all publicly available journal publications. Supplementary Table 1 provides detailed information on the PubMed search strategy.

### 2.3 Research inclusion and exclusion criteria

Articles involving acupuncture and related therapies were eligible, provided that the intervention subjects met the established criteria for VCI. Eligible study types included clinical randomized controlled trials, animal studies, systematic reviews, meta-analyses, and case reports. Studies that did not pertain to acupuncture interventions specifically aimed at VCI were systematically excluded. The criteria ensured that only those studies with a clear focus on the intervention and target population were retained.

### 2.4 Screening and selection of studies

The screening and selection process commenced with the use of EndNote X9 to manage the retrieved literature. Initially, the built-in duplication tool was utilized to eliminate duplicate records, followed by manual checks to remove any remaining duplicates not detected by the software. Subsequently, a thorough review of titles and abstracts was conducted to exclude studies unrelated to acupuncture interventions for VCI. The remaining studies were then subjected to a blinded evaluation by two independent reviewers. Any discrepancies or disagreements encountered during the review process were resolved through consultation with a senior researcher, whose expertise ensured that the final selection was both accurate and consistent.

### 2.5 Extraction of effective data and evidence synthesis

We primarily extracted acupoint information from these studies and compiled it into a table. Additionally, we reclassified the acupoints mentioned in these articles based on the nerves within the anatomical structures underlying each acupoint. The neuroanatomical correlations of the acupuncture points will be referenced from the Chinese higher education “14th Five-Year Plan” textbook *Acupoint Anatomy* (Shao, 2023) and the *Interactive Medical Acupuncture Anatomy* (Teton NewMedia, USA, 2016) (Robinson, 2016). Finally, we categorized and summarized the research progress on acupuncture treatment for VCI based on classifying peripheral nerves and explored the underlying mechanisms involved in these pathways.

## 3 Results

### 3.1 Screening and selection of studies

**Figure 1:**
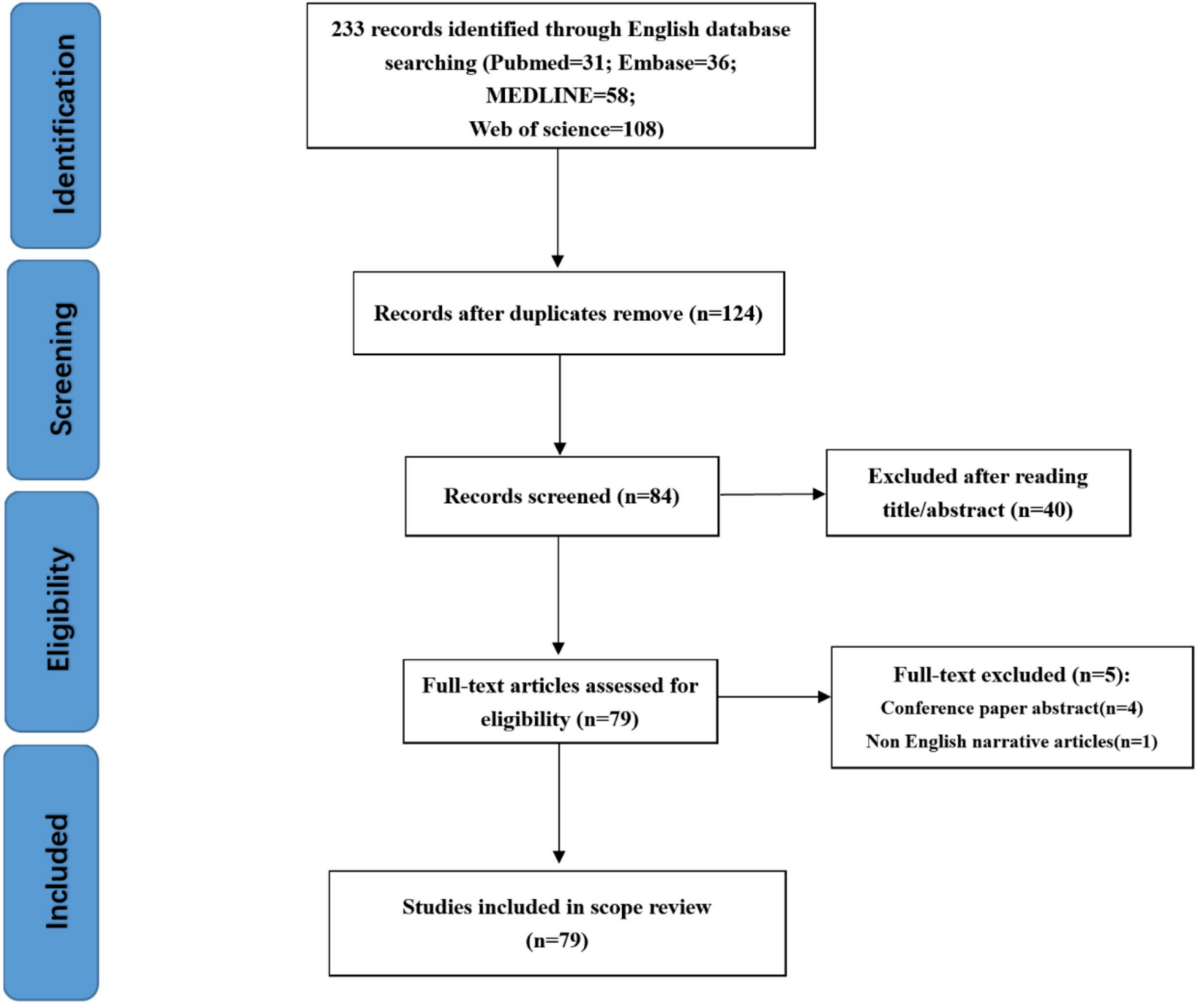
Literature screening and inclusion process.

Through a search of English-language databases, including PubMed, Embase, MEDLINE, and Web of Science, a total of 233 articles were identified (PubMed = 31, Embase = 36, MEDLINE = 58, Web of Science = 108). After removing duplicates, 124 articles remained for the initial screening phase. Of these, 40 articles were excluded based on screening titles and abstracts, leaving 79 articles for full-text evaluation. During the full-text review, two conference abstracts were excluded, resulting in 79 articles that were ultimately included in this review (Figure 1).

### 3.2 Basic characteristics of articles

The reviewed articles cover a timeline from 2005 to 2024, providing a comprehensive overview of research on acupuncture and its effects on VCI and related conditions. The body of research was composed of 32 experimental animal studies, 14 clinical studies (including 12 randomized controlled trials and 2 non-randomized trials), and 33 literature-based investigations. The animal studies were conducted to explore the mechanisms underlying acupuncture’s effects on cognitive function, often employing rat models of multi-infarct dementia or VD to mimic clinical conditions.

On the clinical side, the randomized controlled trials focused on evaluating the efficacy of acupuncture in improving cognitive function in patients with VCI or other forms of cognitive decline. Furthermore, a substantial portion of the literature consists of comprehensive reviews and syntheses of existing data. Among the 33 literature investigations, 31 are systematic reviews, with 13 of these incorporating meta-analyses to quantitatively assess the efficacy of acupuncture. In addition to these rigorous analyses, there is one narrative review that offers a succinct overview of the field, as well as one Delphi consensus study that gathers expert opinions to guide future research directions. Specific information of all literatures can be found in Supplementary Table 2.

### 3.3 Common acupoints and nerves involved

**Figure 2:**
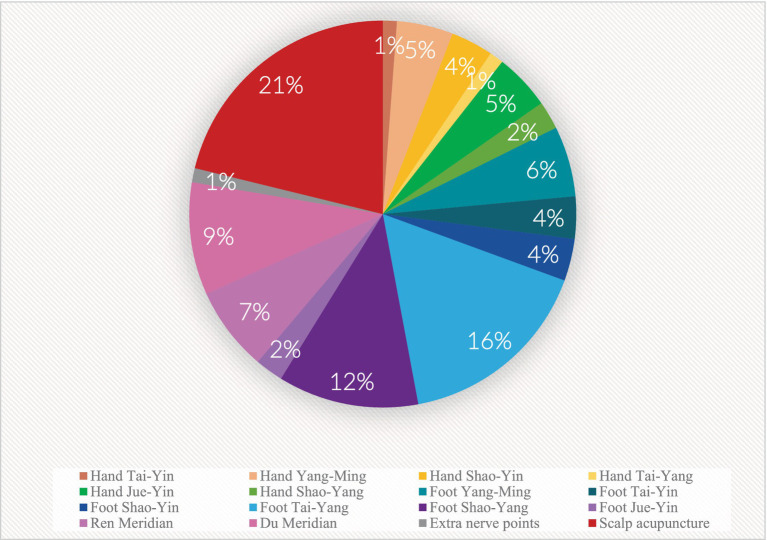
Number of different meridian acupoints.

**Figure 3:**
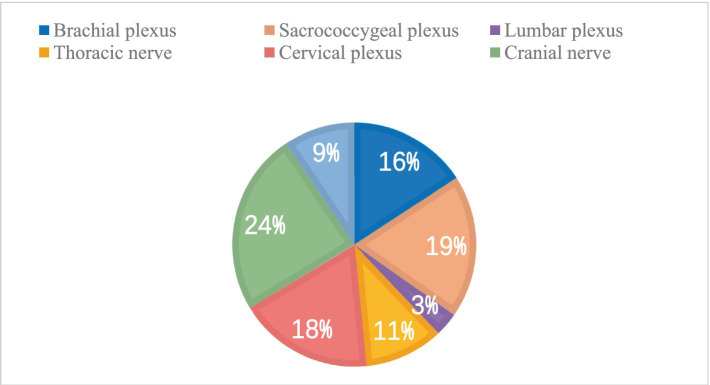
Number of acupoints involved in different nerves.

Based on the statistical analysis of acupoints that appeared across all the included studies, we have summarized the acupoints incorporated across all studies under consideration. All fourteen meridians are represented, including extra nerve acupoints and scalp acupoints. As illustrated in Figure 2, The most commonly involved acupoints are often located in the Taiyang Bladder Meridian, followed by the Foot Shaoyang Gallbladder Meridian and the Governor Vessel. We have categorized the acupoints according to the involved nerves, dividing them into groups that encompass acupoints related to upper limb neural networks and their branches, as well as those pertaining to the lower limbs and sacral region innervated by the lumbosacral plexus and its branches. Other categories include acupoints related to the lower limbs and lumbar-dorsal areas innervated by the lumbar plexus and its branches, as well as back-shu points associated with the thoracic nerve plexus and its branches, neck and occipital acupoints connected to the cervical plexus and its branches, facial and cranial acupoints linked to cranial nerves and their branches, and acupoints distributed along the autonomic nervous system. Notably, cranial nerves, lumbosacral plexus, and cervical plexus acupoints are the most prevalent (Figure 3), with specific acupoints and their corresponding neural affiliations detailed in the Table 1.

**Table 1:** Commonly used acupoints and their related peripheral nerves.

Main neural stems and origins involved	Branching nerve	Involving acupoints
Brachial plexus	Musculocutaneous nerve	LU11 (ShaoShang), LI11 (Quchi), HT7 (ShenMen),HT5 (TongLi),PC6 (NeiGuan), PC5 (JianShi), SJ5 (WaiGuan)
	Radial nerve	LU11 (ShaoShang), LI1 (ShangYang), LI4 (Hegu), SJ5 (WaiGuan), LI11 (QuChi)
	Ulnar nerve	HT7(ShenMen), HT5(TongLi), HT9 (ShaoChong), SJ1 (GuanChong), SI1 (ShaoZe)
	Median nerve	PC6 (NeiGuan), PC5 (JianShi), PC7(DaLing), LU11 (ShaoShang), LI1 (ShangYang), LI4 (Hegu), PC9 (ZhongCong)
Sacrococcygeal plexus	Sciatic/tibial nerve branches	SP6 (ShanYinJiao), KID3(TaiXi), KID4 (DaZhong), KID6 (ZhaoHai), ST36 (ZuSanLi), GB44 (ZuQiaoYin), KID1 (YongQuan), UB67 (ZhiYin), GB30 (HuanTiao)
	Sciatic nerve/common peroneal nerve branch	ST36 (ZuSanLi), GB39 (XuanZhong), LIV3 (TaiChong), LIV1 (DaDun), ST45 (LiDui), SP1 (YinBai), ST40 (FengLong), GB30 (HuanTiao)
	Coccygeal nerve	DU1 (ChangQiang)
Lumbar plexus	Femoral nerve	SP10 (XueHai)
	L3 ~ L4	UB23 (ShenShu), DU3 (YaoYangGuan)
Thoracic plexus	T4	REN17 (DanZhong)
	T5 ~ T6	UB15 (XinShu), DU9 (ZhiYang), UB45(YiXi), DU9 (ZhiYang)
	T7 ~ T8	REN12 (ZhongWan)
	T9 ~ T10	UB18 (GanShu)
	T11 ~ T12	UB20 (PiShu), REN4 (GuanYuan), REN6 (QiHai)
Cervical plexus	Cervical nerve	DU16 (FengFu), DU14 (DaZhui), DU15 (YaMen), Occipital scalp needle, Temporal scalp needle
	Greater occipital nerve	DU16 (FengFu), UB9 (YuZhen), UB10 (TianZhu), GB20 (FengChi), GB8 (ShaiGu), DU20 (BaiHui), DU15 (YaMen), DU17 (NaoHu), DU18 (QiangJian), EX-HN1 (SiShenCong)
	Lesser occipital nerve	GB20 (FengChi), GB12(WanGu), EX-HN13 (YiMing)
Cranial nerve	Trigeminal nerve	ST8 (TouWei), GB8 (ShuaiGu), GB7 (QuBing), EX-HN1 (SiShenCong), DU23 (ShangXing), DU24 (ShenTing), GB14 (YangBai), UB4 (QuChai), DU20 (BaiHui), EX-HN03 (YinTang), LI20 (YingXiang), GB13 (BenShen), GB15 (TouLinQi), Top forehead scalp needle
	Facial nerve	LI20 (YingXiang)
	Auditory nerve	EX-HN13(YiMing)
	Accessory nerve	EX-HN13(YiMing)
	Vagus	EX-HN13(YiMing), Auricular acupoint
Autonomic nerve	Sympathetic nerve	UB23 (ShenShu), DU3 (YaoYangGuan), UB15 (XinShu), DU9 (ZhiYang), UB45 (YiXi), UB18 (GanShu), UB20 (PiShu)
	Parasympathetic nerve	EX-HN13(YiMing), Auricular acupoint

**Figure 4:**
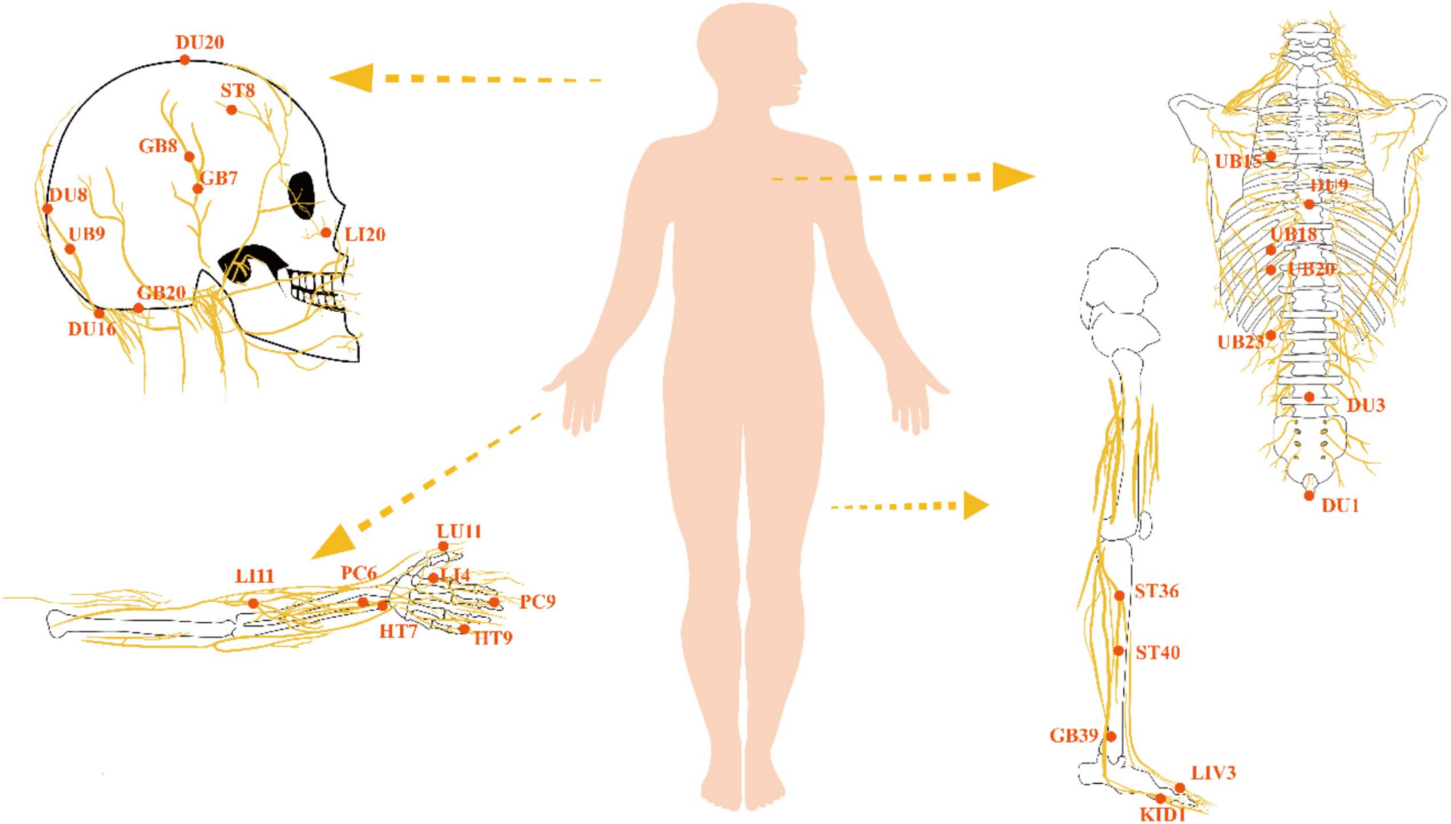
Neural distribution figure of acupoints.

Our examination of acupoints, categorized by meridians and associated neural networks, reveals their predominant distribution around the peripheral nerves of the limbs, cranial nerves, spinal nerves, and the autonomic nervous system. In 1971, Jiao creatively established sixteen stimulation areas on the scalp. Some studies suggest that scalp acupuncture in specific zones can treat injuries to the corresponding brain regions and even induce molecular changes (Sun et al., 2020). Scalp acupoints predominantly project onto cranial and cervical nerves (Dorsher and Chiang, 2018). Additionally, a subset of the summarized acupoints involves the autonomic nervous system. It is established that acupuncture significantly influences the central autonomic regulatory center, a controller of the autonomic nervous system, as demonstrated by a wealth of animal studies (Li et al., 2022; Ohsawa et al., 1995; Uchida et al., 2010; Sato et al., 1993). Acupuncture’s impact on the autonomic nervous system follows a segmental distribution pattern, with the ability to modulate sympathetic/parasympathetic output. Acupuncture engages in diverse neural regulations, and the following sections will categorize them by different neural systems, exploring the pathways and mechanisms through which acupuncture exerts its effects on VCI (Figure 4).

### 3.4 Acupuncture and brachial plexus stimulation

Peripheral nerves are richly distributed in the body’s meridians and acupoints, and the involvement of the peripheral nervous system is essential for the efficacy of acupuncture. Acupuncture can improve cognitive function by stimulating the brachial plexus nerve, typically through the synergistic action of multiple nerve branches. Acupoints commonly used in VCI acupuncture interventions include LI4 (Hegu), PC5 (Jianshi), PC6 (Neiguan), and Waiguan, traversed by the radial nerve, ulnar nerve, median nerve, and musculocutaneous nerve, respectively. The brachial plexus nerve constitutes a complex anatomical nerve network, including branches such as the musculocutaneous nerve, radial nerve, ulnar nerve, median nerve, axillary nerve, thoracodorsal nerve, and long thoracic nerve, primarily supplying the upper limbs (Kattan and Borschel, 2011; Orebaugh and Williams, 2009). An interaction exists between the brachial plexus nerve and cognitive function. The hippocampus, an essential limbic system component, is believed to play a significant role in brain memory and emotional formation processes. Preclinical evidence suggests that chronic peripheral nerve injuries reduce neurogenesis in the adult hippocampus’s granule cell layer of the dentate gyrus (DG) (Vania et al., 2016; Xia et al., 2020). Clinical studies have found activation of the medial-hypothalamic pathway following brachial plexus nerve injury (BPI), a critical structure for pain processing integration (Wang et al., 2019). Changes occur in other brain regions related to cognitive functions post-brachial plexus nerve injury (p-BPI), with decreased functional connectivity within the primary motor cortex (M1) (Fraiman et al., 2016). Cognitive functions participate in the sensory (Mahmoudaliloo et al., 2011) and functional (De Azevedo et al., 2018) recovery processes of the hand after upper limb peripheral nerve injury, where patients with BPI exhibit dynamic changes in the resting-state brain networks (RSNs) during treatment. Patients with improved muscle strength show significantly increased connectivity in the sensorimotor network (SMN) and salience network (SN) post-treatment. In contrast, all patients exhibit a trend of decreased connectivity in the default mode network (DMN) postoperatively, indicating the role of brain plasticity and compensatory mechanisms in the recovery process of peripheral nerve injury (Bhat et al., 2017). Research analyzing the conduction function of peripheral nerves in elderly individuals with different cognitive levels has found that the conduction velocity of the median motor nerve and the peroneal motor nerve differs significantly (Qian et al., 2022), suggesting damage to fast-conducting fibers related to movement in cognitive impairment patients, with a decline in motor conduction ability. Additionally, the motor conduction velocity of the median and peroneal nerve is positively correlated with the ideographic part of the Montreal Cognitive Assessment (MoCA) and verbal fluency task (VFT) (Amira et al., 2024). Magnetic stimulation of PC6 (Neiguan) and PC (Daling) in healthy subjects shows increased functional connectivity of brain areas associated with higher cognitive functions such as emotion, memory, and language (Dai et al., 2019), with activation of brain regions closely related to cognitive and emotional processes, such as the frontal lobe and temporal lobe (Haili et al., 2022), and significant improvement in information transmission efficiency in the brain cortex during stimulation. Acupuncture at LI4 (Hegu) has been shown to modulate frontal and temporal lobe activity in patients with AD and MCI, while enhancing the connectivity between the hippocampus and the motor cortex (Zheng et al., 2018; Wang et al., 2012). Functional magnetic resonance imaging (fMRI) scans of 24 healthy individuals show that cerebellar activation is typically only observed during acupuncture at the Waiguan acupoint (Wik et al., 2016). Traditionally associated with motor coordination and balance, the cerebellum plays a crucial role in various aspects of higher functions, with emerging research revealing its broader contributions to cognition, emotion, and reward processes (Buckner, 2013; Schmahmann, 2019; Wagner and Luo, 2020). Electrical stimulation of the ulnar nerve at an *α* frequency (10 Hz) in Brain-Computer Interface (BCI) subjects increases the classification accuracy of left and right-hand motor imagery from 66.41 to 81.57%, with effects lasting for at least two days (Zhang et al., 2020). Stimulation of the ulnar nerve was considered that can improve motor imagery classification accuracy, enhancing the neural function of cognitive impairment patients.

### 3.5 Acupuncture and lumbo/sacral plexus stimulation

The lumbosacral plexus, originating from L1-S3 spinal nerve roots, controls lower limb sensation and motor function (Di Benedetto et al., 2005). Anatomically, it consists of the “upper” lumbar plexus and the “lower” sacral plexus (Laughlin and Dyck, 2013). The lumbar plexus is located within the psoas major muscle and comprises the anterior branches of the T12 to L4 nerve roots. The nerve branches of the lumbar plexus mainly include the iliohypogastric nerve, ilioinguinal nerve, genitofemoral nerve, lateral femoral cutaneous nerve, obturator nerve, and femoral nerve. The sacral plexus comprises the lumbosacral trunk (L4, L5), all sacral nerves, and the anterior branches of the coccygeal nerve, which originate from the ventral edges of the L4 to S4 nerve roots. The nerve branches of the sacral plexus mainly include the lumbosacral trunk, superior gluteal nerve, inferior gluteal nerve, sciatic trunk/nerve, and pudendal nerve. More research is needed on the relationship between sciatic nerve injury and cognitive impairment among the sacral plexus nerves. A cross-sectional study from China found that as a branch of the sciatic nerve, the peroneal nerve exhibits decreased nerve conduction velocity in patients with MCI and AD, and the conduction velocity of the peroneal nerve is significantly correlated with the diagnosis of AD (Qian et al., 2022; Qian and Xiao, 2020). In addition, sciatic nerve injury can lead to neuropathic pain (Jones et al., 2018), and patients with neuropathic pain often report adverse emotional and cognitive consequences (Mendes et al., 2008; Begoña et al., 2018), such as anxiety, depression, sleep disorders (Radat et al., 2013; Attal et al., 2011), attention deficits, memory difficulties, and decreased decision-making ability (María and Rosa, 2005; Mccracken and Iverson, 2001).

Currently, cognitive function is clinically improved by stimulating the nerve branches of the lumbosacral plexus, which is still in the exploratory stage (Qu et al., 2016; Yamada et al., 2004). However, acupuncture at acupoints where the lumbosacral plexus nerves are distributed can improve cognitive function, usually through the synergistic action of multiple nerve branches. The sciatic nerve is the most commonly involved pathway. Acupoints such as SP6 (Sanyinjiao), KID3 (Taixi), KI1 (Yongquan), and GB30 (Huantiao) are mainly innervated by the branches of the sciatic nerve. Acupoints such as ST36 (Zusanli), LR3 (Taichong), and GB34 (Yanglingquan) are associated with the branches of the sciatic nerve. Evidence suggests that long-term acupoint massage (6 months) can improve cognitive function and activities of daily living in patients with mild to moderate dementia (Liang Ooi et al., 2023), with SP6 being a commonly used acupoint for intervention.

#### 3.5.1 Neural plasticity

Animal experiments have shown that electroacupuncture (EA) at SP6 improves spatial learning ability in rats with daytime cognitive dysfunction after insomnia, possibly related to the upregulation of brain-derived neurotrophic factor (BDNF)/Tropomyosin receptor kinase B (TrkB) signaling and inhibition of neuronal apoptosis after EA (Lina et al., 2023). When acupuncture was applied to rats with ketamine-induced cognitive impairment (Miao et al., 2023), it was found that the rats’ neurological and behavioral symptoms improved, and the neurodegeneration of the locus coeruleus (LC) was alleviated, possibly due to EA regulating the Calmodulin-Dependent Protein Kinase II (CAMKII)/cAMP Response Element-Binding Protein (CREB) pathway, thus mitigating the adverse effects mentioned above. When mice experience cognitive changes due to social isolation, stress-induced increases in neurotrophic factors such as nerve growth factor (NGF) or BDNF occur (Aloe et al., 2002; Alleva et al., 1996), and EA can reverse these changes (Manni et al., 2009). Acupuncture at ST36 significantly reduces the escape latency of VD rats (Li et al., 2015), improves impaired hippocampus-dependent memory in animals, and is associated with regulating energy metabolism in astrocytes, increasing neuron counts, and upregulating synaptic plasticity (Li et al., 2015). Acupuncture at ST36 can enhance calcium signals in pyramidal neurons and astrocytes in the somatosensory cortex, leading to delayed calcium transients in astrocytes, activating neurons and astrocytes (Chang et al., 2022).

#### 3.5.2 Changes in EEG mechanism and brain functional connectivity

Acupuncture at ST36 (Zusanli) modulates both periodic (delta, alpha) and aperiodic (slope, offset) electroencephalogram (EEG) features, reflecting neural entrainment and network changes (Yu et al., 2024; Yu et al., 2018; Yu et al., 2024). Graph theory and machine learning reveal that varied acupuncture techniques produce distinct connectivity patterns, enabling precise classification (Yu et al., 2019; Rao et al., 2025). Resting-state fMRI studies have shown that acupuncture at KI3 (Taixi) not only activates brain regions associated with perception, movement, cognition, association, hearing, and vision in healthy young adults, healthy elderly individuals, and patients with MCI, but also enhances connectivity between the dorsolateral/medial prefrontal cortex and the posterior temporal lobe (Zhu et al., 2015; Chen et al., 2014). Notably, compared with healthy individuals, acupuncture at KI3 (Taixi) in MCI patients can modulate additional cognition-related brain regions: it not only reduces the amplitude of low-frequency fluctuations (ALFF) in areas such as the posterior cingulate gyrus, right frontal lobe, and cerebellum, but also increases ALFF values in the left precuneus, thereby exerting a more significant therapeutic effect under pathological conditions (Jia et al., 2015). Meanwhile, acupuncture at GB34 can regulate local homogeneity in areas including the anterior insula as well as the frontal and temporal lobes (Chen et al., 2012; Feng et al., 2012; Liu et al., 2018). Moreover, fMRI research has found that acupuncture at LR3 primarily activates brain functional networks associated with vision, association, and emotional cognition (Zheng et al., 2016). Further experiments indicate that simultaneous needling of LR3 and KI3 (Taixi) in healthy volunteers activates brain regions related to vision, emotion, and cognition while inhibiting areas linked to emotion, attention, speech semantics, and memory (Zhang et al., 2015).

### 3.6 Acupuncture and cervical plexus stimulation

The cervical plexus comprises the anterior branches of the first to fourth cervical nerves, situated deep to the upper part of the sternocleidomastoid muscle, anterior to the origins of the scalene muscles and levator scapulae muscle (Usui et al., 2010). The primary cutaneous branches of the cervical plexus are (I) the lesser occipital nerve, originating from the C2 spinal nerve, distributed over the posterior aspect of the scalp and ear, providing skin sensation; (II) the transverse nerve of the neck. Composed of branches from C2 and C3, distributed over the anterior neck skin, providing skin sensation; (III) the supraclavicular nerve, formed by the combination of branches from C3 and C4, primarily distributed over the lower lateral neck, upper chest wall, and shoulder skin; (IV) the greater occipital nerve, composed of branches from C2 and C3, distributed over the scalp and adjacent skin. All of these nerves are cutaneous nerves. In addition, the cervical plexus gives off some muscular branches to supply the muscles of the neck region and diaphragm. The phrenic nerve, a motor branch of the cervical plexus, originates from the anterior branches of C3-C5 and provides motor innervation to the diaphragm (Waxenbaum et al., 2024; Kim et al., 2018; Paraskevas et al., 2014). Skin electrodes target the greater occipital nerve, establishing a communication gateway from the periphery to the brain via specific afferent fibers that project to the brainstem and synapse with the nucleus tractus solitarius (NTS) (Luckey et al., 2023; Adair et al., 2020), further activating the LC noradrenergic system of the brainstem, projecting to both the primary cortex and subcortical areas (Berridge and Waterhouse, 2003).

Branches of the cervical nerve, greater occipital nerve, and lesser occipital nerve are distributed under the acupoint of GB20 (Fengchi). Clinical studies have shown that massage of GB20 (Fengchi) and similar acupoints for 6 months can improve cognitive function and activities of daily living in patients with mild to moderate dementia (Liang Ooi et al., 2023). There is also a tiny occipital nerve distributed under the GB12 (Wangu) acupoint. Stimulation of this acupoint significantly improved learning ability in rats compared to the VD model group, with decreased mRNA expression levels of TNF-*α*, IL-6, and IL-1*β* (Fang and Sui, 2016). Animal experiments with laser acupuncture at DU20 (Baihui) have shown a significant increase in neuronal density in the CA1 and CA3 regions, increased activity of glutathione peroxidase (GSH-Px) and superoxide dismutase (SOD), decreased IL-6 and β-amyloid density ratio, exerting antioxidant and anti-inflammatory effects, thereby alleviating cognitive impairment in focal ischemic rats (Jittiwat, 2019). EA at DU20 (Baihui) and DU14 (Dazhui) enhances learning and memory abilities in rats, downregulating levels of inflammatory factors such as IL-1β, JAK2, and TNF-α (Han et al., 2017), alleviating hippocampal synaptic plasticity damage (Zheng et al., 2016), upregulating mRNA expression of BDNF and vascular endothelial growth factor (VEGF) (Kim et al., 2014), increasing regional cerebral blood flow (rCBF) (Han et al., 2017), promoting the regeneration of oligodendrocytes (Ahn et al., 2016), and increasing the number of proliferating cells and differentiated cells in the hippocampus and subventricular zone (Kim et al., 2014). Neuroimaging evidence indicates that EA at DU20 (Baihui) induces an increase in regional homogeneity (ReHo) in multiple areas, including the orbitofrontal cortex (OFC), midcingulate cortex (MCC), precentral cortex, and precuneus. It decreases ReHo values in the anterior cingulate cortex (ACC), supplementary motor area, thalamus, and cerebellum and decreases ReHo values in the precentral gyrus (preCUN) (Yu et al., 2019). Acupuncture at DU20 (Baihui), GB20 (Fengchi), and the Chorea-Tremor Controlled Zone reduced PD tremor by modulating the cerebello-thalamo-cortical circuit (decreased DC/ReHo and increased ALFF) and altering cognitive networks (DMN, visual areas, insula, PFC) (Li et al., 2018).

### 3.7 Acupuncture and thoracic plexus stimulation

Thoracic nerves originate from the thoracic spinal cord. After exiting through the intervertebral foramen, they divide into anterior, posterior, meningeal, and communicating branches. Except for the first and parts of the twelfth pair, the anterior branches do not form plexuses and maintain segmental characteristics. The first to eleventh pairs are intercostal nerves, while the twelfth pair forms the subcostal nerves (Cramer, 2014).

Acupuncture or moxibustion at single or multiple points corresponding to the distribution of thoracic nerves can improve cognitive function. The location of the CV4 (Guanyuan) acupoint corresponds to the peripheral nerves of the T11-T12 segments. Studies have shown that moxibustion at CV4 (Guanyuan) and moxa smoke exposure both ameliorated cognitive deficits in APP/PS1 mice by normalizing tricarboxylic acid cycle flux and unsaturated fatty acid metabolism (Ha et al., 2019). Before the decline in cognitive abilities, there is a significant decrease in cerebral glucose metabolism (Shoshan-Barmatz et al., 2018). Neuronal functional activity requires a lot of energy, which is mainly provided by mitochondria. Mitochondrial defects can lead to synaptic dysfunction, neuroinflammation, and neuronal death (Arnold and Finley, 2023). Peripheral nerves from the T7-T8 segments innervate CV12 (Zhongwan). Using resting-state fMRI, researchers observed brain function changes during EA at CV4 (Guanyuan) and CV12 (Zhongwan) in 21 healthy volunteers. Stimulation significantly enhanced local connectivity in the ventromedial prefrontal cortex network, including the medial orbitofrontal and ventral anterior cingulate cortices, compared to the resting state (Fang et al., 2012).

### 3.8 Acupuncture and trigeminal nerve stimulation

The trigeminal nerve, the fifth cranial nerve, is located on the ventrolateral surface of the brainstem at the midpoint level. It comprises a thicker sensory root and a thinner motor root. Originating from the petrous part of the temporal bone, it terminates in the principal and spinal nuclei within the brainstem. The trigeminal nerve has three main branches—ophthalmic, maxillary, and mandibular—extensively distributed across the eye, forehead, nose, upper jaw, oral cavity, and cheek, and it innervates facial sensation and transmits sensory information from the dura mater (Kumada et al., 1977). It connects to the vasomotor center of the brainstem, including the lateral tegmental area, and is easily stimulated percutaneously (Goadsby et al., 1996; DeGiorgio et al., 2011).

Acupuncture at certain head and facial acupoints often coincide with the trigeminal nerve distribution. For example, ST7 (Xiaguan) is located at the branches of the facial and auriculotemporal nerves, with the mandibular nerve, including the inferior alveolar and lingual nerves. Other points like GB14 (Yangbai) and BL2 (Zanzhu) are distributed with the supraorbital and frontal nerves. According to meridian theory, the trigeminal nerve’s branches partial consistency with the routes of the three yang meridians of the hand and foot. The therapeutic effects of acupuncture at head acupoints seem to be associated with more inputs from the trigeminal nerve and the unique roles of the TG and trigeminal spinal nucleus (STN) in regulating meningeal function (Moskowitz et al., 1998). It is well-known that endogenous substances change after acupuncture. As a neuroregulatory therapy, trigeminal nerve stimulation is believed to treat VCI by increasing dopamine release in the hippocampus through the TG - corticotropin-releasing hormone (CRH) - dopamine transporter (DAT) - hippocampus (HPC) pathway, thereby enhancing hippocampal-dependent memory (Xu et al., 2023). Electrical acupuncture has been repeatedly shown to have effects on neuroprotection (Liu et al., 2017; Shi et al., 2018), synaptic plasticity (Gerrow and Triller, 2010), and regulation of neural cross-talk (Tu et al., 2019; Chang et al., 2018).

#### 3.8.1 Neural plasticity

Following electrical acupuncture stimulation of the trigeminal nerve branches EX-HN3 (Yintang) and DU20 (Baihui), there was a reversal of ischemia–reperfusion injury, with increased expression of BDNF, tyrosine kinase B, N-methyl-D-aspartate receptor 1, alpha-amino-3-hydroxy-5-methyl-4-isoxazolepropionic acid receptor, gamma-aminobutyric acid A receptor, Ca2+/calmodulin-dependent protein kinase II, and synaptic density protein 95 in the prefrontal cortex and hippocampus, leading to improved spatial and cognitive memory in rats (Zheng et al., 2020).

#### 3.8.2 Regulation of cerebral blood flow and cerebral perfusion

Stimulation of the trigeminal nerve has a relevant effect on the cerebral arterial vascular system, increasing CBF through retrograde evoked potentials, trigeminal parasympathetic reflex, and other central pathways (White et al., 2021). Long-term changes in CBF and perfusion are among the causes of VCI. A study found that EA at GB15 (Toulinqi) significantly increased CBF in rats, suggesting a trigeminal nerve-meninges-cerebrospinal fluid pathway as a potential shortcut for brain regulation and treatment (Wang et al., 2017). Electrical stimulation of the trigeminal nerve can balance disturbances in cerebral perfusion (Shiflett et al., 2015; Chiluwal et al., 2017), which is associated with activation of the parasympathetic nervous system (Branston, 1995) and release of related vascular active molecules such as acetylcholine (Ach), vasoactive intestinal peptide (VIP), and nitric oxide (NO) (Shelukhina et al., 2017; Goadsby and Shelley, 1990; Jones et al., 2001). A study found that 100 Hz trigeminal EA enhances parasympathetic tone, upregulates eNOS-derived NO and acetylcholine vasodilation, and improves prefrontal cortical perfusion (Waki et al., 2017). A recent study implanted a neuromodulatory device into the pterygopalatine ganglion located deep beneath the ST7 (Xiaguan) acupoint in patients with acute ischemic stroke (Bornstein et al., 2019). This device stimulation increased CBF and perfusion within 24 h, improving cortical involvement. The effect within 3 h was comparable to intravenous alteplase and exceeded alteplase between 3–4.5 h.

#### 3.8.3 Changes in brain functional connectivity

International standard scalp acupuncture involving the distribution of the trigeminal nerve has been shown to enhance the regional homogeneity of the anterior cingulate cortex and medial prefrontal gyrus in healthy elderly individuals (Chung et al., 2019). Moreover, studies have demonstrated that EA at GV24 (Shenting) and ST8 (Touwei) enhances the functional connectivity of the executive control, sensorimotor, and attention networks, while reducing the connectivity of the DMN (Chen et al., 2023). In addition, among acute ischemic stroke (AIS) patients with high-risk factors for VCI, those who received scalp acupuncture showed significant improvements in NIHSS scores and increased functional connectivity measures (VMHC, ALFF, ReHo) in regions responsible for sensory integration, language processing, and motor coordination (Liu et al., 2021).

### 3.9 Acupuncture and Vagus nerve stimulation

The vagus nerve (VN) is the tenth cranial nerve, with extensive distribution throughout the body, making it the longest and most widely distributed nerve among the cranial nerves. It originates from the medulla oblongata of the brainstem, passes through the jugular foramen into the carotid sheath, travels along the posterior groove between the carotid artery or internal carotid artery and internal jugular vein to reach the root of the neck, and then extends to the thorax and abdomen (Foley and DuBois, 1937). It comprises approximately 20% efferent fibers and 80% afferent fibers. Efferent or motor visceral pathways innervate organs below the neck, including the heart, lungs, and gastrointestinal tract, with the brain receiving information from the afferent projections of the vagus nerve. Afferent fibers project to the NTS and LC within the brainstem (Nomura and Mizuno, 1984), then directly or indirectly project to many brain areas, such as the midbrain, hypothalamus, amygdala, hippocampus, and frontal lobe (Lange et al., 2011; Carreno and Frazer, 2016). The auricular branch of the VN is the only afferent branch located on the body surface, innervating the skin around the external auditory meatus, inner ear pinna, and earlobe (Butt et al., 2020; Kiyokawa et al., 2014; Peuker and Filler, 2002). Moreover, the earlobe is entirely innervated by the auricular branch of the vagus nerve (Peuker and Filler, 2002). It contains approximately 80% of afferent fibers that transmit sensory information to the central nervous system, while the remaining fibers transmit motor information. A recent systematic review suggests (Robinson et al., 2012) that the auricular branch of the vagus nerve projects to the NTS, which further connects with other brain regions such as LC, lateral hypothalamus, amygdala, anterior cingulate cortex, insular cortex, and ventral tegmental area.

The EX-HN13 (YiMing) acupoint and auricular acupoint are commonly used acupoints in clinical practice for treating VCI. Stimulating these acupoints may activate pathways related to the vagus nerve. The EX-HN13 (YiMing) acupoint is located 1 cun posterior to TB-17 (YiFeng) at the inferior border of the mastoid process. The YiMing acupoint’s deep needling can stimulate the vagus nerve’s trunk. Some ear acupoints, such as heart (concha, CO15) and kidney (CO10), are widely distributed with the auricular vagus nerve. It is well-known that electrical stimulation of the vagus nerve and transcutaneous electrical stimulation of the auricular vagus nerve (taVNS) are two therapies that are quite similar in principle and mechanism of action. taVNS likely originated in China, with traditional Chinese medicine theory considering the relationship between “ear acupoints” and “disease” based on meridian theory and holistic concepts (Pj et al., 2015). In 1990, the World Health Organization recognized ear acupuncture as a microsystem of acupuncture that can positively affect systemic function regulation (World Health Organization, 1990).

#### 3.9.1 Neural plasticity

The occurrence of VCI is closely related to central nervous system damage caused by ischemia and hemorrhage, and neuroplasticity is important for the recovery of cognitive function. VNS can upregulate the expression of BDNF and dendritic spines in the hippocampus (Biggio et al., 2009) and can activate serotonergic neurons in the hippocampus (Manta et al., 2009). Neurotransmitters associated with VNS, such as norepinephrine released from the LC that activates adrenergic receptors, play a crucial role in enhancing attention, reactivity, and other cognitive functions (Cheng et al., 2022). Ach supports memory formation and gamma-aminobutyric acid (GABA) not only inhibits but also coordinates excitatory activity, both playing key roles in cognitive enhancement (Trifilio et al., 2023). Electrical current pulses at a certain frequency for ear stimulation can upregulate the activation of the locus coeruleus-norepinephrine pathway, and upregulate the release of acetylcholine and GABA (Trifilio et al., 2023).

#### 3.9.2 Anti-inflammation mechanism

Neuroinflammation is an immune cascade reaction mediated by glial cells in the central nervous system where innate immunity resides. Various destructive events, including hypoxia, ischemia, and infection, can trigger inflammatory responses. Long-term chronic hypoperfusion-induced ischemia and hypoxia can over-activate neuroinflammation, leading to pathological changes such as cell apoptosis and BBB damage, exacerbating the occurrence and development of cognitive impairment (Tian et al., 2022). The parasympathetic ganglia in the vagus nerve express receptors for interleukin IL-1 (Sternberg, 2006), and communication between the afferent and efferent branches of the vagus nerve in the NTS constitutes a connection between somatic immunity and the central nervous system (Thayer and Sternberg, 2010). The NTS projects to the dorsal motor nucleus of the vagus nerve (DMV) and nucleus ambiguous (NA), where these signals regulate many immune organs such as the spleen, liver, and gastrointestinal tract, while acetylcholine released by the vagus nerve can regulate immune responses by modulating various cell receptors such as NF-κB’s α7 nicotinic acetylcholine receptor (α7nAChR) (Ma et al., 2019), these parasympathetic neural pathways together constitute the so-called “cholinergic anti-inflammatory pathway.” Furthermore, the regulation of microglia by the vagus nerve is also considered relevant to cognition, as degeneration of the LC and downregulation of norepinephrine levels in the projection area can lead to increased activation of microglia and astrocytes (Kaczmarczyk et al., 2017). The chronic activation and polarization of microglia can activate inflammatory responses. VNS can restore microglia to a healthy state by upregulating norepinephrine through the LC, and changes in microglial expression can regulate neurotrophic factors such as BDNF and bFGF, as well as anti-inflammatory cytokines such as IL-4, IL-10, and TGF-*β*, thereby adjusting the tendency of neuroinflammation (Clancy et al., 2014).

#### 3.9.3 Regulations of cerebrospinal fluid circulation, cerebral blood flow, and perfusion

Cerebrospinal fluid circulation dysfunction is associated with cognitive impairment, and patients with cognitive impairment may experience cerebrospinal fluid circulation disorders, which correlate with symptoms’ severity (Attier-Zmudka et al., 2019). A study on the relationship between VNS and cerebrospinal fluid circulation reported that VNS after stroke-induced VCI can enhance cerebrospinal fluid circulation (Cheng et al., 2020), which is beneficial for the metabolism and excretion of toxic metabolites in the brain substance during the progression of ischemic stroke, such as amyloid-beta and Nogo-A. Reduced CBF leading to brain damage is also an important condition for the onset of VCI, and vascular remodeling is crucial in the course of stroke and cerebral small vessel disease. The establishment of collateral circulation and angiogenesis are the main reasons for increasing cerebral blood volume (Liu et al., 2014). VNS has been shown to increase microvascular density and endothelial cell proliferation around the ischemic area (Jiang et al., 2016), and upregulate the expression of BDNF, VEGF (Jiang et al., 2016), and GDF11 (Katsimpardi et al., 2014), which can promote angiogenesis and increase the proliferative capacity of brain capillary endothelial cells (ECs). Other studies have also shown that VNS may act by increasing CBF in the bilateral thalamus, hypothalamus, cerebellar hemisphere, right postcentral gyrus, frontal lobe cortex, and cingulate cortex (Conway et al., 2006).

#### 3.9.4 Changes in brain functional connectivity

With the development of functional magnetic resonance imaging, brain functional connectivity is another major approach to treating VCI. VNS transmits signals to important brain areas, such as the LC and NTS, through electrical pulse conduction (Schachter and Saper, 1998). These structures relay signals to higher structures such as the hippocampus, insula, frontal lobe cortex, and motor cortex (Dolphin et al., 2022). The LC, as the noradrenergic (NE) center, is believed to play an important role in cognitive function, with its LC-NE system associated with attention, executive function, memory, and emotion recognition (Aston-Jones and Cohen, 2005). VNS is believed to be associated with this (Naparstek et al., 2023). Another theory suggests that cognitive aging and reduced cerebral hemisphere activity, such as in the frontal lobe cortex (Dolcos et al., 2002), to some extent exacerbate this barrier. VNS as an additional neural circuit can be a compensatory neural resource to enhance cognitive function (Reuter-Lorenz and Cappell, 2008).

#### 3.9.5 Other effects

Some studies have also proposed that VNS can reduce BBB permeability and protect its integrity. During the onset of VCI, the integrity of the BBB is beneficial for preventing harmful fluids, chemicals, and blood-derived cells from entering the brain parenchyma, thereby reducing the incidence of inflammatory reactions (Jiang et al., 2018). Additionally, during ischemia–reperfusion injury, VNS can downregulate the levels of TUNEL-positive cells and cleaved caspase-3 protein in the ischemic penumbra and upregulate p-Akt to inhibit the occurrence of cell apoptosis (Jiang et al., 2014). VNS can also affect cognition through the brain-gut axis pathway, which is related to the autonomic regulation function of the vagus nerve (Carabotti et al., 2015). The enteric nervous system (ENS) can produce more than 30 neurotransmitters, which can be released into the bloodstream through the BBB. Communication between the vagus nerve and ENS is mainly mediated by cholinergic activation, serving as a flow of information between the gut and the vagus nerve and central nervous system (Carabotti et al., 2015; Schemann, 2005).

### 3.10 Acupuncture and other nerves stimulation

Several potential acupuncture mechanisms involve other cranial nerves, such as the facial, optic, and olfactory nerves. Multiple facial acupoints involve the distribution of the facial nerve, such as ST7 (Xiaguan), which distributes the zygomaticotemporal branch of the facial nerve and fiber projections from the facial nerve nucleus. Facial nerve stimulation has been studied for increasing CBF in ischemic stroke (Borsody et al., 2013; Borsody et al., 2014). A study explored the minimum stimulation parameters for increasing CBF with facial nerve magnetic stimulation in Yorkshire pigs and tested safety, tolerability, and efficacy in healthy volunteers (Sanchez et al., 2018). The results indicated that continuous stimulation of the facial nerve at 1.6–1.8 Tesla power for approximately 10 min resulted in an average CBF of 32 ± 6%, with a ≥ 25% increase observed in 10 out of 31 volunteers. Some other cranial nerve pathways are gradually being discovered, such as the optic nerve and olfactory nerve. There is an association between the optic nerve and MCI, as retinal imaging through fundus photographs or optical coherence tomography (OCT) observes changes in the optic nerve head to identify early cognitive impairment (Gao et al., 2023). MCI patients exhibit significant progressive tau pathology and deposition of amyloid plaques in the olfactory area (CAm), with signal changes from the olfactory nerve possibly predicting the development of dementia (Bathini et al., 2019). Neural stimulation not only affects a series of changes in the peripheral and central systems but also heralds changes in the function of higher centers with changes in peripheral nerve signals. The nervous system operates like a precise, tightly linked network, where changes in any link may lead to effects on the periphery or even central nervous system changes. Many nerves, such as the optic nerve and olfactory nerve, cannot be stimulated through acupuncture, suggesting that improvements or new inventions are anticipated.

## 4 Discussion

The neural pathway is one of the main avenues for achieving acupuncture effects, especially in treating peripheral and central nervous system diseases. Stimulating different nerve trunks may have different effects, as the types of nerve fibers contained in nerve trunks vary, with a bundle of nerves potentially containing up to seven different types of nerve fibers. These nerve fibers include somatic afferent and efferent nerve fibers and autonomic nerve fibers. Stimulation of a certain nerve trunk may evoke multiple and diverse nerve reflex effects, and with the varying number and complexity of different nerve fibers, the effects and pathways become even more intricate. For central nervous system diseases such as VCI, cerebrovascular accidents, and AD, neural stimulation therapy may be an effective and direct approach, as it circumvents the restrictive effects of the BBB on certain drugs. Unraveling the mechanisms underlying the central effects induced by nerve stimulation reflexes is conducive to advancing the renewal of existing therapies. And it holds significant importance in revealing the mechanisms of acupuncture.

**Figure 5:**
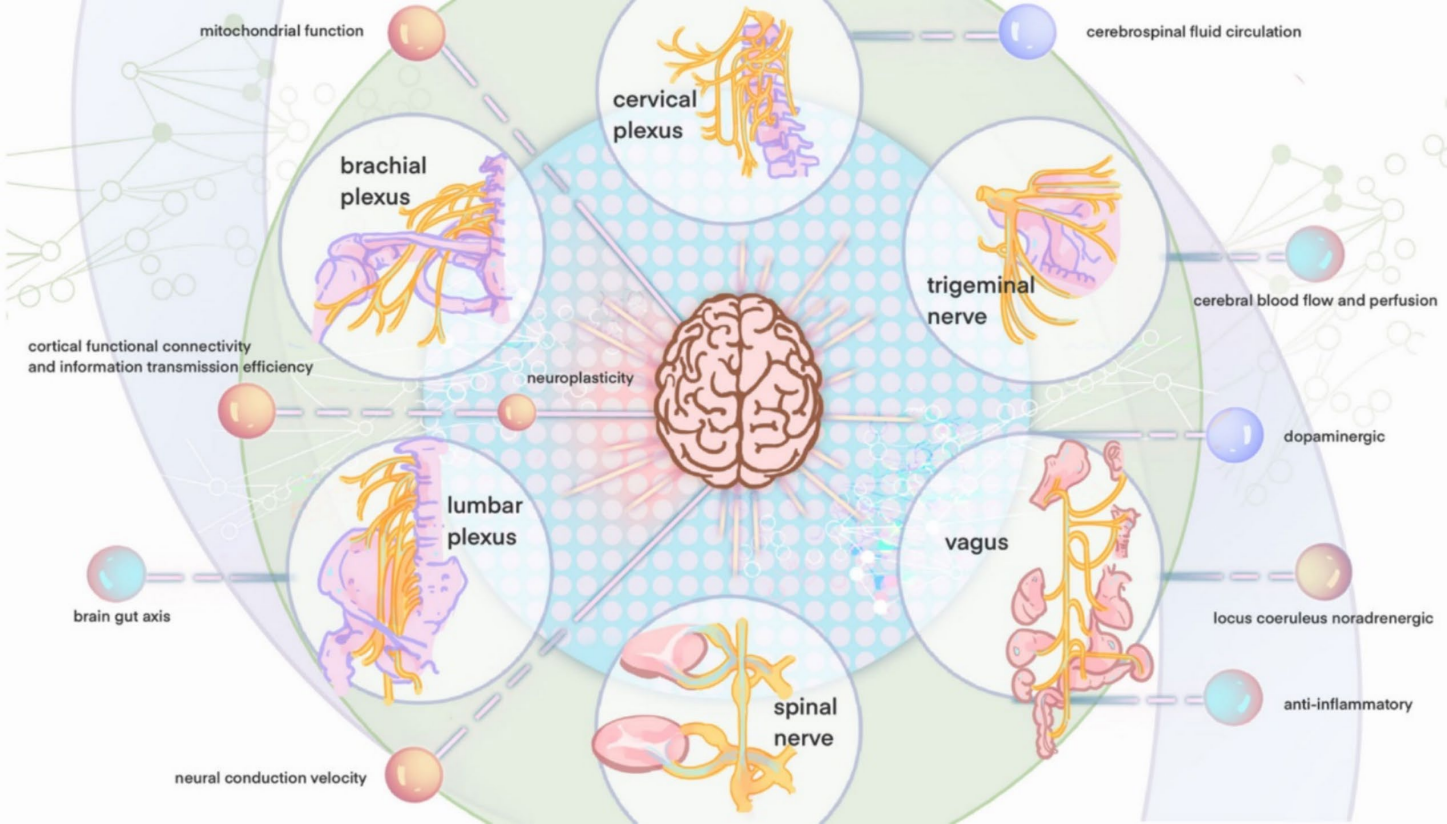
The central effect mechanism of peripheral nerve stimulation.

When attempting to unravel the complex neural effects of acupuncture on central nervous system diseases such as VCI based on different nerve types, it is found that the differences in acupuncture effects based on acupoints are indeed related to anatomical and neural structural variations. For instance, somatic afferent and efferent nerve stimulation pathways are more closely associated with central nervous system plasticity and brain functional connectivity. In contrast, the autonomic nervous pathway is more relevant to anti-inflammatory responses and CBF regulation (Figure 5). The brachial plexus via LI4 (Hegu) and PC6 (Neiguan) enhances cognitive function by modulating hippocampal connectivity, increasing motor cortex activity, and improving motor imagery accuracy through median and ulnar nerve stimulation. EA at PC6 (Neiguan) upregulates BDNF while suppressing axon-growth inhibitors, thereby promoting neuronal sprouting and synaptogenesis (Mu et al., 2023). Lumbosacral plexus stimulation at SP6 and Zusanli (ST36) improves spatial learning, alleviates neuropathic pain, and boosts mitochondrial function via BDNF/TrkB signaling. Zusanli also increases VEGF, activating PI3K/ERK pathways to foster neurogenesis and cortical angiogenesis and translating into enhanced synaptic plasticity and functional recovery (Mu et al., 2023). Cervical plexus activation at GB20 (Fengchi) and Baihui (DU20) reduces inflammation, enhances antioxidant activity, and modulates both sensorimotor and default mode networks. Stimulation of Baihui elevates BDNF and VEGF in ischemic brain tissue and improves cerebral perfusion and repair through angiogenesis-driven mechanisms (Li et al., 2022). Thoracic nerve stimulation at CV4 (Guanyuan) and CV12 (Zhongwan) enhances prefrontal connectivity and cerebral glucose metabolism. Trigeminal nerve activation at EX-HN3 (Yintang) and GB15 (Toulinqi) increases CBF, upregulates synaptic proteins such as BDNF, and combats ischemia–reperfusion injury. VNS at EX-HN3 (Yintang) and auricular points promotes anti-inflammatory responses via the cholinergic pathway in which vagal efferents act on macrophage α7 nicotinic acetylcholine receptors to reduce systemic inflammation (Oh and Kim, 2022). This stimulation also upregulates neurotransmitters including norepinephrine and acetylcholine and enhances both angiogenesis and cerebrospinal fluid circulation. Emerging evidence suggests that facial and optic nerve stimulation may influence cerebral perfusion and serve as early biomarkers of cognitive change. Together these neuroplastic and anti-inflammatory mechanisms provide a coherent basis for acupuncture’s therapeutic effects on cognitive function.

However, there some limitations in our review. First, our literature search used limited search terms and databases, potentially missing relevant studies. This incomplete retrieval could bias our conclusions by overlooking contradictory or supportive evidence. Second, we did not perform a meta-analysis or quantitative synthesis, limiting our ability to accurately estimate effect sizes or consistency across studies; thus, our findings remain qualitative. Third, attributing specific mechanisms to individual acupoints is based largely on indirect evidence, inferred from general findings or animal studies rather than confirmed by direct experimental data. Fourth, many reviewed studies employed combined acupoint protocols rather than testing single points separately. Finally, research on somatic nerve involvement in cognitive impairment remains limited, restricting our understanding of how sensory or motor signal deficits may contribute to neuronal loss and cognitive decline. Therefore, targeted studies exploring isolated acupoint mechanisms and somatic nerve roles in cognitive disorders are needed.

Future research should address these gaps by focusing on specific acupoint mechanisms through targeted single-acupoint studies. This approach could clarify the unique molecular pathways associated with each acupoint. Additionally, integrating multimodal methods such as functional MRI or EEG combined with molecular biomarkers (e.g., BDNF, inflammatory cytokines) would enhance our understanding of how acupuncture-induced neural changes correlate with therapeutic outcomes. Further systematic exploration of EA parameters, including frequency and intensity, could optimize therapeutic efficacy by identifying conditions that specifically activate desired neural pathways. Finally, translating these findings into rigorously designed clinical trials is essential to validate mechanisms observed in animal models. Long-term human studies could assess whether acupuncture confers sustained neuroplastic or disease-modifying benefits, thereby advancing its evidence-based clinical application. Of course, as unknown territories emerge, the principles of acupuncture, an “ancient therapy,” and the scientific connotations of meridians and acupoint theories will be further revealed.

## Data Availability

The original contributions presented in the study are included in the article/Supplementary material, further inquiries can be directed to the corresponding authors.

## References

[ref1] Adair D. Truong D. Esmaeilpour Z. Gebodh N. Borges H. Ho L. . (2020). Electrical stimulation of cranial nerves in cognition and disease. Brain Stimul. 13, 717–750. doi: 10.1016/j.brs.2020.02.019 32289703 PMC7196013

[ref2] Ahn S. M. Kim Y. R. Kim H. N. Shin Y. I. Shin H. K. Choi B. T. (2016). Electroacupuncture ameliorates memory impairments by enhancing oligodendrocyte regeneration in a mouse model of prolonged cerebral hypoperfusion. Sci. Rep. 6:28646. doi: 10.1038/srep28646 27350403 PMC4923909

[ref3] Alleva E. Petruzzi S. Cirulli F. Aloe L. (1996). Ngf regulatory role in stress and coping of rodents and humans. Pharmacol. Biochem. Behav. 54, 65–72. doi: 10.1016/0091-3057(95)02111-6 8728540

[ref4] Aloe L. Alleva E. Fiore M. (2002). Stress and nerve growth factor: findings in animal models and humans. Pharmacol. Biochem. Behav. 73, 159–166. doi: 10.1016/S0091-3057(02)00757-8 12076735

[ref5] Alzheimers Disease International. World Alzheimer report 2018: The state of the art of dementia research: New frontiers. (2018). Available online at: https://www.alzint.org/resource/world-alzheimer-report-2018/ (Accessed August 20, 2021).

[ref6] Amira A. L. Ahmed H. R. Lamis S. M. Ahmed H. M. Nawal A. Ashraf A. D. (2024). Relationship between diabetic peripheral neuropathy and cognitive functions. Med. J. Cairo Univ. 91, 1641–1646. doi: 10.21608/mjcu.2024.342785

[ref7] Arksey H. O’Malley L. (2005). Scoping studies: towards a methodological framework. Int. J. Soc. Res. Methodol. 8, 19–32. doi: 10.1080/1364557032000119616

[ref8] Arnold P. K. Finley L. W. S. (2023). Regulation and function of the mammalian tricarboxylic acid cycle. J. Biol. Chem. 299:102838. doi: 10.1016/j.jbc.2022.102838 36581208 PMC9871338

[ref9] Aston-Jones G. Cohen J. (2005). An integrative theory of locus coeruleusnorepinephrine function: adaptive gain and optimal performance. Annu. Rev. Neurosci. 28, 403–450. doi: 10.1146/annurev.neuro.28.061604.135709 16022602

[ref10] Attal N. Lanteri-Minet M. Laurent B. Fermanian J. Bouhassira D. (2011). The specific disease burden of neuropathic pain: results of a French nationwide survey. Pain 152, 2836–2843. doi: 10.1016/j.pain.2011.09.014 22019149

[ref11] Attier-Zmudka J. Sérot J.-M. Valluy J. Saffarini M. Macaret A.-S. Diouf M. . (2019). Decreased cerebrospinal fluid flow is associated with cognitive deficit in elderly patients. Front. Aging Neurosci. 11:87. doi: 10.3389/fnagi.2019.00087 31114494 PMC6502902

[ref12] Bathini P. Mottas A. Jaquet M. Brai E. Alberi L. (2019). Progressive signaling changes in the olfactory nerve of patients with Alzheimer’s disease. Neurobiol. Aging 76, 80–95. doi: 10.1016/j.neurobiolaging.2018.12.006 30708185

[ref13] Battle C. E. Abdul-Rahim A. H. Shenkin S. D. Hewitt J. Quinn T. J. (2021). Cholinesterase inhibitors for vascular dementia and other vascular cognitive impairments: a network meta-analysis. Cochrane Database Syst. Rev. 2:Cd013306. doi: 10.1002/14651858.CD013306.pub2 33704781 PMC8407366

[ref14] Begoña O. María D. Alejandro S. . (2018). Factors influencing cognitive impairment in neuropathic and musculoskeletal pain and fibromyalgia. Pain Med. 19, 499–510. doi: 10.1093/pm/pnx024 28340167

[ref15] Berridge C. W. Waterhouse B. D. (2003). The locus coeruleus–noradrenergic system: modulation of behavioral state and state-dependent cognitive processes. Brain Res. Rev. 42, 33–84. doi: 10.1016/S0165-0173(03)00143-7 12668290

[ref16] Bhat D. I. Indira Devi B. Bharti K. Panda R. (2017). Cortical plasticity after brachial plexus injury and repair: a resting-state functional Mri study. Neurosurg. Focus. 42:E14. doi: 10.3171/2016.12.FOCUS16430 28245732

[ref17] Biesbroek J. M. Kuijf H. J. van der Graaf Y. Vincken K. L. Postma A. Mali W. . (2013). Association between subcortical vascular lesion location and cognition: a voxel-based and tract-based lesion-symptom mapping study. The smart-Mr study. PLoS One 8:e60541. doi: 10.1371/journal.pone.0060541 23593238 PMC3620525

[ref18] Biggio F. Gorini G. Utzeri C. Olla P. Marrosu F. Mocchetti I. . (2009). Chronic vagus nerve stimulation induces neuronal plasticity in the rat hippocampus. Int. J. Neuropsychopharmacol. 12, 1209–1221. doi: 10.1017/S1461145709000200 19309534 PMC2879889

[ref19] Bikson M. Grossman P. Thomas C. Zannou A. L. Jiang J. Adnan T. . (2016). Safety oftranscranial direct current stimulation: evidence basedupdate 2016. Brain Stimul. 9, 641–661. doi: 10.1016/j.brs.2016.06.004 27372845 PMC5007190

[ref20] Bornstein N. M. Saver J. L. Diener H. C. Gorelick P. B. Shuaib A. Solberg Y. . (2019). Impact-24B investigators. An injectable implant to stimulate the sphenopalatine ganglion for treatment of acute ischaemic stroke up to 24 h from onset (impact-24B): an international, randomised, double-blind, sham-controlled, pivotal trial. Lancet 394, 219–229. doi: 10.1016/S0140-6736(19)31192-4 31133406

[ref21] Borsody M. K. Yamada C. Bielawski D. Heaton T. Castro Prado F. Garcia A. . (2014). Effects of noninvasive facial nerve stimulation in the dog middle cerebral artery occlusion model of ischemic stroke. Stroke 45, 1102–1107. doi: 10.1161/STROKEAHA.113.003243 24549865

[ref22] Borsody M. K. Yamada C. Bielawski D. Heaton T. Lyeth B. Garcia A. . (2013). Effect of pulsed magnetic stimulation of the facial nerve on cerebral blood flow. Brain Res. 1528, 58–67. doi: 10.1016/j.brainres.2013.06.022 23850647

[ref23] Branston N. M. (1995). The physiology of the cerebrovascular parasympathetic innervation. Br. J. Neurosurg. 9, 319–330. doi: 10.1080/02688699550041331 7546354

[ref24] Buckner R. L. (2013). The cerebellum and cognitive function: 25 years of insight from anatomy and neuroimaging. Neuron 80, 807–815. doi: 10.1016/j.neuron.2013.10.044 24183029

[ref25] Butt M. F. Albusoda A. Farmer A. D. Aziz Q. (2020). The anatomical basis for transcutaneous auricular vagus nerve stimulation. J. Anat. 236, 588–611. doi: 10.1111/joa.13122 31742681 PMC7083568

[ref26] Carabotti M. Scirocco A. Ma M. Severi C. (2015). The gut-brain axis: interac tions between enteric microbiota, central and enteric nervous systems. Ann. Gastroenterol. 28, 203–209, PMID: 25830558 PMC4367209

[ref27] Carreno F. R. Frazer A. (2016). The allure of transcutaneous vagus nerve stimulation as a novel therapeutic modality. Biol. Psychiatry 79, 260–261. doi: 10.1016/j.biopsych.2015.11.016 26796874

[ref28] Chang X. Y. Chen K. Cheng T. Lai P. T. Zhang L. So K. F. . (2022). In vivo neuronal and astrocytic activation in somatosensory cortex by acupuncture stimuli. Neural Regen. Res. 17, 2526–2529. doi: 10.4103/1673-5374.339003 35535906 PMC9120680

[ref29] Chang Q.-Y. Lin Y.-W. Hsieh C.-L. (2018). Acupuncture and neuroregeneration in ischemic stroke. Neural Regen. Res. 13, 573–583. doi: 10.4103/1673-5374.230272, PMID: 29722298 PMC5950656

[ref30] Chen H. Jann K. Li Y. Huang J. Chen Y. Kang Y. . (2023). A true response of the brain network during electroacupuncture stimulation at scalp acupoints: an fmri with simultaneous Eas study. Brain Behav. 13:e2829. doi: 10.1002/brb3.2829 36427258 PMC9847615

[ref31] Chen S. J. Meng L. Yan H. Bai L. J. Wang F. Huang Y. . (2012). Functional organization of complex brain networks modulated by acupuncture at different acupoints belonging to the same anatomic segment. Chin. Med. J. 125, 2694–2700. doi: 10.3760/cma.j.issn.0366-6999.2012.15.009 22931977

[ref32] Chen S. Xu M. Li H. Liang J. Yin L. Liu X. . (2014). Acupuncture at the Taixi (Ki3) acupoint activates cerebral neurons in elderly patients with mild cognitive impairment. Neural Regen. Res. 9, 1163–1168. doi: 10.4103/1673-5374.135319 25206776 PMC4146092

[ref33] Cheng K. P. Brodnick S. K. Blanz S. L. Zeng W. Kegel J. Pisaniello J. A. . (2020). Clinically-derived vagus nerve stimulation enhances cerebrospinal fluid penetrance. Brain Stimul. 13, 1024–1030. doi: 10.1016/j.brs.2020.03.012 32388045

[ref34] Cheng K. Wang Z. Bai J. Xiong J. Chen J. Ni J. (2022). Research advances in the application of vagus nerve electrical stimulation in ischemic stroke. Front. Neurosci. 16:1043446. doi: 10.3389/fnins.2022.1043446 36389255 PMC9650138

[ref35] Chiluwal A. Narayan R. K. Chaung W. Mehan N. Wang P. Bouton C. E. . (2017). Neuroprotective effects of trigeminal nerve stimulation in severe traumatic brain injury. Sci. Rep. 7:6792. doi: 10.1038/s41598-017-07219-3 28754973 PMC5533766

[ref36] Cho Y. Han Y. Kim Y. Han S. Oh K. Chae H. . (2022). Anatomical structures and needling method of the back-shu points Bl18, Bl20, and Bl22 related to gastrointestinal organs: a Prisma-compliant systematic review of acupoints and exploratory mechanism analysis. Medicine 101:e29878. doi: 10.1097/MD.0000000000029878 36316824 PMC9622668

[ref37] Choi S. Jang D. C. Chung G. Kim S. K. (2022). Transcutaneous auricular Vagus nerve stimulation enhances cerebrospinal fluid circulation and restores cognitive function in the rodent model of vascular cognitive impairment. Cells 11:3019. doi: 10.3390/cells11193019 36230988 PMC9564197

[ref38] Chung W. Y. Liu S. Y. Gao J. C. Jiang Y. J. Zhang J. Qu S. S. . (2019). Modulatory effect of international standard scalp acupuncture on brain activation in the elderly as revealed by resting-state fmri. Neural Regen. Res. 14, 2126–2131. doi: 10.4103/1673-5374.262590 31397351 PMC6788231

[ref39] Clancy J. A. Mary D. A. Witte K. K. Greenwood J. P. Deuchars S. A. Deuchars J. (2014). Non-invasive vagus nerve stimulation in healthy humans reduces sympathetic nerve activity. Brain Stimul. 7, 871–877. doi: 10.1016/j.brs.2014.07.031 25164906

[ref40] Conway C. R. Sheline Y. I. Chibnall J. T. George M. S. Fletcher J. W. Mintun M. A. (2006). Cerebral blood flow changes during vagus nerve stimulation for depression. Psychiatry Res. 146, 179–184. doi: 10.1016/j.pscychresns.2005.12.007 16510266

[ref41] Corriveau R. A. Bosetti F. Emr M. Gladman J. T. Koenig J. I. Moy C. S. . (2016). The science of vascular contributions to cognitive impairment and dementia (VCID): a framework for advancing research priorities in the cerebrovascular biology of cognitive decline. Cell. Mol. Neurobiol. 36, 281–288. doi: 10.1007/s10571-016-0334-7 27095366 PMC4859348

[ref42] Cramer G. D. (2014). The thoracic region. Clinical Anatomy of the Spine, Spinal Cord, and Ans. 210–245. doi: 10.1016/B978-0-323-07954-9.00006-2

[ref43] Dai Y. Y. Yin N. Yu H. . (2019). Cerebral cortex functional networks of magnetic stimulation at acupoints along the pericardium meridian. J. Integr. Neurosci. 18, 79–85. doi: 10.31083/j.jin.2019.01.126, PMID: 31091852

[ref44] Dangour A. D. Allen E. Elbourne D. Fasey N. Fletcher A. E. Hardy P. . (2010). Efect of 2-y n-3 long-chain polyunsaturated fatty acid supplementation on cognitive function in older people: a randomized, double-blind, controlled trial. Am. J. Clin. Nutr. 91, 1725–1732. doi: 10.3945/ajcn.2009.29121 20410089

[ref45] De Azevedo F. A. S. Santos W. Z. De Oliveira T. G. Abdouni Y. A. Costa A. C. D. Fucs P. M. (2018). Does cognitive capacity interfere with the outcome of Oberlin transfer? Acta Ortopedica Brasileira 26, 394–396. doi: 10.1590/1413-785220182606196665 30774513 PMC6362679

[ref46] DeGiorgio C. M. Fanselow E. E. Schrader L. M. Cook I. A. (2011). Trigeminal nerve stimulation: seminal animal and human studies for epilepsy and depression. Neurosurg. Clin. N. Am. 22, 449–456. doi: 10.1016/j.nec.2011.07.001 21939843

[ref47] Di Benedetto P. Pinto G. Arcioni R. De Blasi R. A. Sorrentino L. Rossifragola I. . (2005). Anatomy and imaging of lumbar plexus. Minerva Anestesiol. 71, 549–554, PMID: 16166916

[ref48] Dolcos F. Rice H. J. Cabeza R. (2002). Hemispheric asymmetry and aging: right hemisphere decline or asymmetry reduction. Neurosci. Biobehav. Rev. 26, 819–825. doi: 10.1016/S0149-7634(02)00068-4 12470693

[ref49] Dolphin H. Dukelow T. Finucane C. Commins S. McElwaine P. Kennelly S. (2022). The wandering nerve linking heart and mind- the complementary role of transcutaneous vagus nerve stimulation in modulating neuro-cardiovascular and cognitive performance. Front. Neurosci. 16:897303. doi: 10.3389/fnins.2022.897303 35784842 PMC9245542

[ref50] Dorsher P. T. Chiang P. (2018). Neuroembryology of the acupuncture principal meridians: part 3. The head and neck. Med. Acupunct. 30, 80–88. doi: 10.1089/acu.2018.1271 29682148 PMC5908427

[ref51] Esposito M. F. Malayil R. Hanes M. Deer T. (2019). Unique characteristicsof the dorsal root ganglion as a target for neuromodulation. Pain Med. 20, S23–S30. doi: 10.1093/pm/pnz012 PMC6544557 31152179

[ref52] Fang Y. Sui R. (2016). Electroacupuncture at the Wangu Acupoint suppresses expression of inflammatory cytokines in the Hippocampus of rats with vascular dementia. AJTCAM 13, 17–24. doi: 10.21010/ajtcam.v13i5.3 28487889 PMC5416636

[ref53] Fang J. Wang X. Liu H. Wang Y. Zhou K. Hong Y. . (2012). The limbic-prefrontal network modulated by Electroacupuncture at Cv4 and Cv12. Evid. Based Complement. Alternat. Med. 2012:515893. doi: 10.1155/2012/515893 22291848 PMC3265182

[ref54] Feng Y. Bai L. Ren Y. Chen S. Wang H. Zhang W. . (2012). Fmri connectivity analysis of acupuncture effects on the whole brain network in mild cognitive impairment patients. Magn. Reson. Imaging 30, 672–682. doi: 10.1016/j.mri.2012.01.003 22459434

[ref55] Fitzpatrick A. L. Kuller L. H. Ives D. G. Lopez O. L. Jagust W. Breitner J. C. S. . (2004). Incidence and prevalence of dementia in the cardiovascular health study. J. Am. Geriatr. Soc. 52, 195–204. doi: 10.1111/j.1532-5415.2004.52058.x 14728627

[ref56] Foley J. O. DuBois F. S. (1937). Quantitative studies of the vagus nerve in the cat. I. The ratio of sensory to motor fibers. J. Comp. Neurol. 67, 49–67. doi: 10.1002/cne.900670104

[ref57] Fraiman D. Miranda M. F. Erthal F. Buur M. Elschot L. Souza S. A. . (2016). Reduced functional connectivity within the primary motor cortex of patients with brachial plexus injury. NeuroImage 12, 277–284. doi: 10.1016/j.nicl.2016.07.008 27547727 PMC4982914

[ref58] Gao H. Zhao S. Zheng G. Wang X. Zhao R. Pan Z. . (2023). Using a dual-stream attention neural network to characterize mild cognitive impairment based on retinal images. Comput. Biol. Med. 166:107411. doi: 10.1016/j.compbiomed.2023.107411 37738896

[ref59] Gerrow K. Triller A. (2010). Synaptic stability and plasticity in a floating world. Curr. Opin. Neurobiol. 20, 631–639. doi: 10.1016/j.conb.2010.06.010 20655734

[ref60] Goadsby P. J. Shelley S. (1990). High-frequency stimulation of the facial nerve results in local cortical release of vasoactive intestinal polypeptide in the anesthetised cat. Neurosci. Lett. 112, 282–289. doi: 10.1016/0304-3940(90)90217-W 1972788

[ref61] Goadsby P. J. Uddman R. Edvinsson L. (1996). Cerebral vasodilatation in the cat involves nitric oxide from parasympathetic nerves. Brain Res. 707, 110–118. doi: 10.1016/0006-8993(95)01206-0 8866719

[ref62] Gong Y. Li N. Lv Z. Zhang K. Zhang Y. Yang T. . (2020). The neuro-immune microenvironment of acupoints-initiation of acupuncture effectiveness. J. Leukoc. Biol. 108, 189–198. doi: 10.1002/JLB.3AB0420-361RR 32645257

[ref63] Ha L. Yu M. Yan Z. Rui Z. Zhao B. (2019). Effects of moxibustion and moxa smoke on behavior changes and energy metabolism in app/Ps1 mice. Evid. Based Complement. Alternat. Med. 2019, 1–10. doi: 10.1155/2019/9419567 PMC6710728 31485251

[ref64] Haili W. Ning Y. Aoxiang W. Xu G. (2022). Cerebral cortex functional networks of transdermal electrical stimulation at Daling (Pc7) Acupoint. Clin. EEG Neurosci. 54:15500594221123692. doi: 10.1177/15500594221123692 36113449

[ref65] Han D. Liu Z. Wang G. Zhang Y. Wu Z. (2017). Electroacupuncture improves cognitive deficits through increasing regional cerebral blood flow and alleviating inflammation in cci rats. Evid. Based Complement. Alternat. Med. 2017:5173168. doi: 10.1155/2017/5173168, PMID: 28491108 PMC5402249

[ref66] Huang M. Wang X. Xing B. Yang H. Sa Z. Zhang D. . (2018). Critical roles of Trpv 2 channels, histamine H1 and adenosine A1 receptors in the initiation of acupoint signals for acupuncture analgesia. Sci. Rep. 8:6523. doi: 10.1038/s41598-018-24654-y 29695862 PMC5916903

[ref67] Jia B. Liu Z. Min B. Wang Z. Zhou A. Li Y. . (2015). The effects of acupuncture at real or sham Acupoints on the intrinsic brain activity in mild cognitive impairment patients. Evid. Based Complem. Alternat. Med. 2015:529675. doi: 10.1155/2015/529675 PMC4433670 26064166

[ref68] Jiang X. Andjelkovic A. V. Zhu L. Yang T. Bennett M. V. L. Chen J. . (2018). Blood-brain barrier dysfunction and recovery after ischemic stroke. Prog. Neurobiol. 163-164, 144–171. doi: 10.1016/j.pneurobio.2017.10.001 28987927 PMC5886838

[ref69] Jiang Y. Li L. Liu B. Zhang Y. Chen Q. Li C. (2014). Vagus nerve stimulation attenuates cerebral ischemia and reperfusion injury via endogenous cholinergic pathway in rat. PLoS One 9:e102342. doi: 10.1371/journal.pone.0102342 25036185 PMC4103831

[ref70] Jiang Y. Li L. Ma J. Zhang L. Niu F. Feng T. . (2016). Auricular vagus nerve stimulation promotes functional recovery and enhances the post-ischemic angiogenic response in an ischemia/reperfusion rat model. Neurochem. Int. 97, 73–82. doi: 10.1016/j.neuint.2016.02.009 26964767

[ref71] Jittiwat J. (2019). Baihui point laser acupuncture ameliorates cognitive impairment, motor deficit, and neuronal loss partly via antioxidant and anti-inflammatory effects in an animal model of focal ischemic stroke. Evid. Based Complement. Alternat. Med. 2019, 1–9. doi: 10.1155/2019/1204709, PMID: 30915140 PMC6409074

[ref72] Jones M. G. Lever I. Bingham S. Read S. McMahon S. Parsons A. (2001). Nitric oxide potentiates response of trigeminal neurones to dural or facial stimulation in the rat. Cephalalgia 21, 643–655. doi: 10.1046/j.1468-2982.2001.00213.x 11531896

[ref73] Jones P. E. Meyer R. M. Faillace W. J. Landau M. E. Smith J. K. McKay P. L. . (2018). Combat injury of the sciatic nerve - an institutional experience. Mil. Med. 183, e434–e441. doi: 10.1093/milmed/usy030 29590419

[ref74] Kaczmarczyk R. Tejera D. Simon B. J. Heneka M. T. (2017). Microglia modulation through external vagus nerve stimulation in a murine model of Alzheimer’s disease. J. Neurochem. 146, 76–85. doi: 10.1111/jnc.14284 29266221

[ref75] Katsimpardi L. Litterman N. K. Schein P. A. Miller C. M. Loffredo F. S. Wojtkiewicz G. R. . (2014). Vascular and neurogenic rejuvenation of the aging mouse brain by young systemic factors. Science 344, 630–634. doi: 10.1126/science.1251141 24797482 PMC4123747

[ref76] Kattan A. E. Borschel G. H. (2011). Anatomy of the brachial plexus. J. Pediatr. Rehabil. Med. 4, 107–111. doi: 10.3233/PRM-2011-0163 21955968

[ref77] Kim Y. R. Kim H. N. Ahn S. M. Choi Y. H. Shin H. K. Choi B. T. (2014). Electroacupuncture promotes post-stroke functional recovery via enhancing endogenous neurogenesis in mouse focal cerebral ischemia. PLoS One 9:e90000. doi: 10.1371/journal.pone.0090000 24587178 PMC3933702

[ref78] Kim J. S. Ko J. S. Bang S. Kim H. Lee S. Y. (2018). Cervical plexus block. Korean J. Anesthesiol. 71, 274–288. doi: 10.4097/kja.d.18.00143 29969890 PMC6078883

[ref79] Kiyokawa J. Yamaguchi K. Okada R. Maehara T. Akita K. (2014). Origin, course and distribution of the nerves to the posterosuperior wall of the external acoustic meatus. Anat. Sci. Int. 89, 238–245. doi: 10.1007/s12565-014-0231-4 24604237

[ref80] Kumada M. Dampney R. A. Reis D. J. (1977). The trigeminal depressor response: a novel vasodepressor response originating from the trigeminal system. Brain Res. 119, 305–326. doi: 10.1016/0006-8993(77)90313-4 830389

[ref81] Lange G. Janal M. N. Maniker A. Fitzgibbons J. Fobler M. Cook D. . (2011). Safety and efficacy of vagus nerve stimulation in fibromyalgia: a phase I/ii proof of concept trial. Pain Med. 12, 1406–1413. doi: 10.1111/j.1526-4637.2011.01203.x 21812908 PMC3173600

[ref82] Laughlin R. S. Dyck P. J. B. (2013). Electrodiagnostic testing in lumbosacral Plexopathies. Phys. Med. Rehabil. Clin. N. Am. 24, 93–105. doi: 10.1016/j.pmr.2012.08.014 23177033

[ref83] Li Z. Chen J. Cheng J. Huang S. Hu Y. Wu Y. . (2018). Acupuncture modulates the Cerebello-Thalamo-cortical circuit and cognitive brain regions in patients of Parkinson’s disease with tremor. Front. Aging Neurosci. 10:206. doi: 10.3389/fnagi.2018.00206 30034336 PMC6043808

[ref84] Li N. Guo Y. Gong Y. Zhang Y. Fan W. Yao K. . (2021). The anti-inflammatory actions and mechanisms of acupuncture from Acupoint to target organs via neuro-immune regulation. J. Inflamm. Res. 14, 7191–7224. doi: 10.2147/JIR.S341581 34992414 PMC8710088

[ref85] Li Y. W. Li W. Wang S. T. Gong Y. N. Dou B. M. Lyu Z. X. . (2022). The autonomic nervous system: a potential link to the efficacy of acupuncture. Front. Neurosci. 16:1038945. doi: 10.3389/fnins.2022.1038945 36570846 PMC9772996

[ref86] Li Q. Q. Shi G. X. Yang J. W. Li Z. X. Zhang Z. H. He T. . (2015). Hippocampal camp/Pka/Creb is required for neuroprotective effect of acupuncture. Physiol. Behav. 139, 482–490. doi: 10.1016/j.physbeh.2014.12.001, PMID: 25481359

[ref87] Li M. Wang Y. Gao Y. Yao X. Lan W. Tang W. (2022). Effects of electroacupuncture on angiogenesis and cortical Vegf and Bdnf expression in rats with focal cerebral ischemia. J. Acupunct. Tuina Sci. 20, 91–103. doi: 10.1007/s11726-022-1300-1

[ref88] Li F. Yan C. Q. Lin L. T. Li H. Zeng X. H. Liu Y. . (2015). Acupuncture attenuates cognitive deficits and increases pyramidal neuron number in hippocampal Ca1 area of vascular dementia rats. BMC Complement. Altern. Med. 15:133. doi: 10.1186/s12906-015-0656-x 25928206 PMC4426171

[ref89] Liang Ooi S. Drew G. Cheon P. S. (2023). Acupressure and dementia: a review of current evidence. Altern. Ther. Health Med. 29, 18–29., PMID: 35427233

[ref90] Lina Q. Yinan S. Lianhong T. . (2023). Efficacy of electroacupuncture stimulating Shenmen (Ht7), Baihui (Du20), Sanyinjiao (Sp6) on spatial learning and memory deficits in rats with insomnia induced by Para-chlorophenylalanine: a single acupoint combined acupoints. J. Trad. Chin. Med. 43, 704–714. doi: 10.19852/j.cnki.jtcm.20230308.001 PMC10320443 37454255

[ref91] Liu L. Chen S. Zeng D. Li H. Shi C. Zhang L. (2018). Cerebral activation effects of acupuncture at Yanglinquan (Gb34) point acquired using resting-state fmri. Comput. Med. Imaging Graph. 67, 55–58. doi: 10.1016/j.compmedimag.2018.04.004 29800886

[ref92] Liu H. Jiang Y. Wang N. Yan H. Chen L. Gao J. . (2021). Scalp acupuncture enhances local brain regions functional activities and functional connections between cerebral hemispheres in acute ischemic stroke patients. Anat. Rec. 304, 2538–2551. doi: 10.1002/ar.24746 PMC9290874 34431612

[ref93] Liu Y. Li C. Wang J. Fang Y. Sun H. Tao X. . (2017). Nafamostat Mesilate improves neurological outcome and axonal regeneration after stroke in rats. Mol. Neurobiol. 54, 4217–4231. doi: 10.1007/s12035-016-9999-7 27335029

[ref94] Liu J. Wang Y. Akamatsu Y. Lee C. C. Stetler R. A. Lawton M. T. . (2014). Vascular remodeling after ischemic stroke: mechanisms and therapeutic potentials. Prog. Neurobiol. 115, 138–156. doi: 10.1016/j.pneurobio.2013.11.004 24291532 PMC4295834

[ref95] Luckey A. M. Adcock K. Vanneste S. (2023). Peripheral nerve stimulation: a neuromodulation-based approach. Neurosci. Biobehav. Rev. 149:105180. doi: 10.1016/j.neubiorev.2023.105180, PMID: 37059406

[ref96] Ma J. Qiao P. Li Q. Wang Y. Zhang L. Lj Y. . (2019). Vagus nerve stimulation as a promising adjunctive treatment for ischemic stroke. Neurochem. Int. 131:104539. doi: 10.1016/j.neuint.2019.104539 31445074

[ref97] Mahmoudaliloo M. Bakhshipour A. Hashemi T. Roofigari A. R. Hassan-Zadeh R. . (2011). The correlation of cognitive capacity with recovery of hand sensibility after peripheral nerve injury of upper extremity. Neuro Rehabil. 29, 373–379. doi: 10.3233/NRE-2011-0715 22207065

[ref98] Manni L. Aloe L. Fiore M. (2009). Changes in cognition induced by social isolation in the mouse are restored by electro-acupuncture. Physiol. Behav. 98, 537–542. doi: 10.1016/j.physbeh.2009.08.011 19733189

[ref99] Manta S. Dong J. Debonnel G. Blier P. (2009). Enhancement of the function of rat serotonin and norepinephrine neurons by sustained vagus nerve stimulation. J. Psychiatry Neurosci. 34, 272–280. doi: 10.1016/j.jpsychires.2009.01.008 19568478 PMC2702444

[ref100] María M. Rosa E. (2005). Reports of memory functioning by patients with chronic pain. Clin. J. Pain 21, 287–291. doi: 10.1097/01.ajp.0000173993.53733.2e 15951644

[ref101] Mccracken L. M. Iverson G. L. (2001). Predicting complaints of impaired cognitive functioning in patients with chronic pain. J. Pain Symptom Manag. 21, 392–396. doi: 10.1016/S0885-3924(01)00267-6 11369160

[ref102] Mendes V. S. M. Oliveira V. A. Lopes D. P. E. Carvalhoo K. M. Rodrigues M. E. S. Sousa Lima R. C. . (2008). The psychophysiology of pain: a literature review. Reciis 2, 85–94. doi: 10.3395/reciis.v2i1.133en

[ref103] Miao H. Li R. Li W. Wu F. Li H. Luo H. (2023). Electroacupuncture attenuates ketamine-induced neuronal injury in the locus coeruleus of rats through modulation of the Camk ii/Creb pathway. Brain Res. Bull. 202:110724. doi: 10.1016/j.brainresbull.2023.110724, PMID: 37543295

[ref104] Moskowitz M. A. Wei E. P. Saito K. I. Kontos H. A. (1998). Trigeminalectomy modifies pial arteriolar responses to hypertension or norepinephrine. Am. J. Phys. 255, H1–H6. 10.1152/ajpheart.1988.255.1.H1 3394814

[ref105] Mu J. D. Ma L. X. Zhang Z. Qian X. Zhang Q. Y. Ma L. H. . (2023). The factors affecting neurogenesis after stroke and the role of acupuncture. Front. Neurol. 14:1082625. doi: 10.3389/fneur.2023.1082625 36741282 PMC9895425

[ref106] NalderL. ZhengB. ChiandetG. MiddletonL. T. de JagerC. A. (2021). Vitamin B12 and folate status in cognitively healthy older adults and associations with cognitive performance. J. Nutr. Health Aging 25, 287–294. doi: 10.1007/s12603-020-1489-y33575718

[ref107] Naparstek S. Yeh A. K. Mills-Finnerty C. (2023). Transcutaneous Vagus nerve stimulation (tvns) applications in cognitive aging: a review and commentary. Front. Aging Neurosci. 15:1145207. doi: 10.3389/fnagi.2023.1145207 37496757 PMC10366452

[ref108] Nitsche M. A. Müller-Dahlhaus F. Paulus W. Ziemann U. (2012). Thepharmacology of neuroplasticity induced by non-invasivebrain stimulation: building models for the clinical use ofcns active drugs. J. Physiol. 590, 4641–4662. doi: 10.1113/jphysiol.2012.232975 22869014 PMC3487028

[ref109] Nomura S. Mizuno N. (1984). Central distribution of primary afferent fibers in the Arnold’s nerve (the auricular branch of the vagus nerve): a transganglionic Hrp study in the cat. Brain Res. 292, 199–205. doi: 10.1016/0006-8993(84)90756-X 6692153

[ref110] Oh J. E. Kim S. N. (2022). Anti-inflammatory effects of acupuncture at St36 point: a literature review in animal studies. Front. Immunol. 12:813748. doi: 10.3389/fimmu.2021.813748 35095910 PMC8790576

[ref111] Ohsawa H. Okada K. Nishijo K. Sato Y. (1995). Neural mechanism of depressor responses of arterial pressure elicited by acupuncturelike stimulation to a hindlimb in anesthetized rats. J. Auton. Nerv. Syst. 51, 27–35. doi: 10.1016/0165-1838(95)80004-T 7722213

[ref112] Orebaugh S. L. Williams B. A. (2009). Brachial plexus anatomy: normal and variant. Sci. World J. 9, 300–312. doi: 10.1100/tsw.2009.39 PMC5823154 19412559

[ref113] Paraskevas G. K. Natsis K. Nitsa Z. Mavrodi A. Kitsoulis P. (2014). Unusual morphological pattern and distribution of the ansa cervicalis: a case report. Roman. J. Morphol. Embryol. 55, 993–996. 25329134

[ref114] Pedditzi E. Peters R. Beckett N. (2016). The risk of overweight/obesity in mid-life and late life for the development of dementia: a systematic review and meta-analysis of longitudinal studies. Age Ageing 45, 14–21. doi: 10.1093/ageing/afv151 26764391

[ref115] Peuker E. T. Filler T. J. (2002). The nerve supply of the human auricle. Clin. Anat. 15, 35–37. doi: 10.1002/ca.1089 11835542

[ref116] Pj R. Jj Z. Yq L. Litscher D. Sy L. Gaischek I. . (2015). Auricular acupuncture and biomedical research – a promising Sino-Austrian research cooperation. Chin. J. Integr. Med. 21, 887–894. doi: 10.1007/s11655-015-2090-9 26631173

[ref117] Qian X. Xiao S. (2020). Peripheral nerve conduction study in early cognitive impairment of Alzheimer’s disease: neuropsychiatry and behavioral neurology/mild cognitive impairment/early symptomatic disease. Alzheimers Dement. 16:e041671. doi: 10.1002/alz.041671

[ref118] Qian X. Yue L. Mellor D. Robbins N. M. Li W. Xiao S. (2022). Reduced peripheral nerve conduction velocity is associated with Alzheimer’s disease: a cross-sectional study from China. Neuropsychiatr. Dis. Treat. 18, 231–242. doi: 10.2147/NDT.S349005 35177907 PMC8846612

[ref119] Qu X. Yan J. Li X. Zhang P. Liu X. (2016). Topography of synchronization of somatosensory evoked potentials elicited by stimulation of the sciatic nerve in rat. Front. Comput. Neurosci. 10:43. doi: 10.3389/fncom.2016.00043 27199728 PMC4854893

[ref120] Radat F. Margot-Duclot A. Attal N. (2013). Psychiatric co-morbidities in patients with chronic peripheral neuropathic pain: a multicentre cohort study. Eur. J. Pain 17, 1547–1557. doi: 10.1002/j.1532-2149.2013.00334.x 23720357

[ref121] Rao W. Xu M. Wang H. Hua W. Guo J. Zhang Y. . (2025). Acupuncture state detection at Zusanli (St-36) based on scalp Eeg and transformer. IEEE J Biomed Health Inform. doi: 10.1109/JBHI.2025.3540924 [ahead of print]. 40031811

[ref122] Ratan R. R. Schiff N. D. (2018). Protecting and repairing the brain. Curr. Opin. Neurol. 31, 669–671. doi: 10.1097/WCO.0000000000000623 30379701

[ref123] Reitz C. Tang M.-X. Manly J. Mayeux R. Ja L. (2007). Hypertension and the risk of mild cognitive impairment. Arch. Neurol. 64, 1734–1740. doi: 10.1001/archneur.64.12.1734 18071036 PMC2672564

[ref124] Reuter-Lorenz P. A. Cappell K. A. (2008). Neurocognitive aging and the compensation hypothesis. Curr. Dir. Psychol. Sci. 17, 177–182. doi: 10.1111/j.1467-8721.2008.00570.x

[ref125] Robinson N. G. (2016). Interactive medical acupuncture anatomy. Jackson, WY: Teton New Media.

[ref126] Robinson N. Lorenc A. Ding W. Jia J. Bovey M. Wang X. (2012). Exploring practice characteristics and research priorities of practitioners of traditional acupuncture in China and the Eu – a survey. J. Ethnopharmacol. 140, 604–613. doi: 10.1016/j.jep.2012.01.052 22338645

[ref127] Saczynski J. S. Jónsdóttir M. K. Garcia M. E. Jonsson P. V. Peila R. Eiriksdottir G. . (2008). Cognitive impairment: an increasingly important complication of type 2 diabetes: the age, gene/environment susceptibility–Reykjavik study. Am. J. Epidemiol. 168, 1132–1139. doi: 10.1093/aje/kwn228 18836152 PMC2727243

[ref128] Sanchez O. García A. Castro-Prado F. Perez M. Lara-Estrada R. Ramirez-Meza M. . (2018). Facial nerve stimulation in normal pigs and healthy human volunteers: transitional development of a medical device for the emergency treatment of ischemic stroke. J. Transl. Med. 16:27. doi: 10.1186/s12967-018-1398-6 29448967 PMC5815230

[ref129] Sato A. Sato Y. Suzuki A. Uchida S. (1993). Neural mechanisms of the reflex inhibition and excitation of gastric motility elicited by acupuncturelike stimulation in anesthetized rats. Neurosci. Res. 18, 53–62. doi: 10.1016/0168-0102(93)90105-Y 8134020

[ref130] Schachter S. C. Saper C. B. (1998). Vagus nerve stimulation. Epilepsia 39, 677–686. doi: 10.1111/j.1528-1157.1998.tb01151.x 9670894

[ref131] Schemann M. (2005). Control of gastrointestinal motility by the “gut brain” – the enteric nervous system. J. Pediatr. Gastroenterol. Nutr. 41, S4–S6. doi: 10.1097/01.scs.0000180285.51365.55, PMID: 16131964

[ref132] Schmahmann J. D. (2019). The cerebellum and cognition. Neurosci. Lett. 688, 62–75. doi: 10.1016/j.neulet.2018.07.005, PMID: 29997061

[ref133] Sdrulla A. D. Guan Y. Raja S. N. (2018). Spinal cord stimulation: clinical efficacy and potential mechanisms. Pain Pract. 18, 1048–1067. doi: 10.1111/papr.12692 29526043 PMC6391880

[ref134] Shao J. (2023). Acupoint Anatomy. Beijing, China: China traditional Chinese medicine press.

[ref135] Shelukhina I. Mikhailov N. Abushik P. Nurullin L. Nikolsky E. E. Giniatullin R. (2017). Cholinergic nociceptive mechanisms in rat meninges and trigeminal ganglia: potential implications for migraine pain. Front. Neurol. 8:163. doi: 10.3389/fneur.2017.00163 28496430 PMC5406407

[ref136] Shi X. Ohta Y. Shang J. Morihara R. Nakano Y. Fukui Y. . (2018). Neuroprotective effects of Smtp-44D in mice stroke model in relation to neurovascular unit and trophic coupling. J. Neurosci. Res. 96, 1887–1899. doi: 10.1002/jnr.24326 30242877

[ref137] Shiflett J. M. Parent A. Golanov E. (2015). Forehead stimulation decreases volume of the infarction triggered by permanent occlusion of middle cerebral artery in rats. J. Neurol. Stroke 2:67. doi: 10.15406/jnsk.2015.02.00067

[ref138] Shoshan-Barmatz V. Nahon-Crystal E. Shteinfer-Kuzmine A. Gupta R. (2018). VDAC 1, mitochondrial dysfunction, and Alzheimer’s disease. Pharmacol. Res. 131, 87–101. doi: 10.1016/j.phrs.2018.03.010, PMID: 29551631

[ref139] Sternberg E. M. (2006). Neural regulation of innate immunity: a coordinated nonspecific response to pathogens. Nat. Rev. Immunol. 6, 318–328. doi: 10.1038/nri1810 16557263 PMC1783839

[ref140] Stough C. Downey L. Silber B. Lloyd J. Kure C. Wesnes K. . (2012). The efects of 90-day supplementation with the omega-3 essential fatty acid docosahexaenoic acid (Dha) on cognitive function and visual acuity in a healthy aging population. Neurobiol. Aging 33, 824.e1–824.e3. doi: 10.1016/j.neurobiolaging.2011.03.019 21531481

[ref141] Sun L. Fan Y. Fan W. Sun J. Ai X. Qiao H. (2020). Efficacy and safety of scalp acupuncture in improving neurological dysfunction after ischemic stroke: a protocol for systematic review and meta-analysis. Medicine 99:e23294. doi: 10.1097/MD.0000000000023294, PMID: 32846808 PMC7447452

[ref142] Thayer J. F. Sternberg E. M. (2010). Neural aspects of immunomodulation: focus on the vagus nerve. Brain Behav. Immun. 24, 1223–1228. doi: 10.1016/j.bbi.2010.07.247 20674737 PMC2949498

[ref143] Tian Z. Ji X. Liu J. (2022). Neuroinflammation in vascular cognitive impairment and dementia: current evidence, advances, and prospects. Int. J. Mol. Sci. 23:224. doi: 10.3390/ijms23116224 PMC9181710 35682903

[ref144] Torres-Rosas R. Yehia G. Peña G. Mishra P. del Rocio Thompson-Bonilla M. Moreno-Eutimio M. A. . (2014). Dopamine mediates vagal modulation of the immune system by electroacupuncture. Nat. Med. 20, 291–295. doi: 10.1038/nm.3479 24562381 PMC3949155

[ref145] Tricco A. C. Lillie E. Zarin W. O’Brien K. K. Colquhoun H. Levac D. . (2018). Prisma extension for scoping reviews (Prisma-ScR): checklist and explanation. Ann. Intern. Med. 169, 467–473. doi: 10.7326/M18-0850 30178033

[ref146] Trifilio E. Shortell D. Olshan S. O’Neal A. Coyne J. Lamb D. . (2023). Impact of transcutaneous vagus nerve stimulation on healthy cognitive and brain aging. Front. Neurosci. 17:1184051. doi: 10.3389/fnins.2023.1184051 37575296 PMC10416636

[ref147] Tu C. H. Mac Donald I. Chen Y. H. (2019). The effects of acupuncture on glutamatergic neurotransmission in depression, anxiety, schizophrenia, and Alzheimer’s disease: a review of the literature. Front. Psych. 10:14. doi: 10.3389/fpsyt.2019.00014, PMID: 30809158 PMC6379324

[ref148] Uchida S. Kagitani F. Hotta H. (2010). Neural mechanisms of reflex inhibition of heart rate elicited by acupuncture-like stimulation in anesthetized rats. Autonomic Neurosci. 157, 18–23. doi: 10.1016/j.autneu.2010.03.021 20460195

[ref149] Usui Y. Kobayashi T. Kakinuma H. Watanabe K. Kitajima T. Matsuno K. (2010). An anatomical basis for blocking of the deep cervical plexus and cervical sympathetic tract using an ultrasound-guided technique. Anesth. Analg. 110, 964–968. doi: 10.1213/ANE.0b013e3181c91ea0 20008914

[ref150] van de Rest O. Geleijnse J. M. Kok F. J. van Staveren W. A. Dullemeijer C. Olderikkert M. G. M. . (2008). Efect of fsh oil on cognitive performance in older subjects: a randomized, controlled trial. Neurology 71, 430–438. doi: 10.1212/01.wnl.0000324268.45138.86 18678826

[ref151] van der Flier W. M. Skoog I. Schneider J. A. Pantoni L. Mok V. Chen C. L. H. . (2018). Vascular cognitive impairment. Nat. Rev. Dis. Primers 4:18003. doi: 10.1038/nrdp.2018.3 29446769

[ref152] Vania A. A. Mutso A. A. Centeno M. V. Kan L. Wu M. Levinstein M. . (2016). Role of adult hippocampal neurogenesis in persistent pain. Pain 157, 418–428. doi: 10.1097/j.pain.0000000000000332 26313405 PMC4858177

[ref153] Wagner M. J. Luo L. (2020). Neocortex-cerebellum circuits for cognitive processing. Trends Neurosci. 43, 42–54. doi: 10.1016/j.tins.2019.11.002 31787351 PMC6942222

[ref154] Waki H. Suzuki T. Tanaka Y. Tamai H. Minakawa Y. Miyazaki S. . (2017). Effects of electroacupuncture to the trigeminal nerve area on the autonomic nervous system and cerebral blood flow in the prefrontal cortex. Acupunct. Med. 35, 339–344. doi: 10.1136/acupmed-2016-011247 28765118

[ref155] Wang S. Liu K. Wang Y. Wang S. He X. Cui X. . (2017). A proposed neurologic pathway for scalp acupuncture: trigeminal nerve-meninges-cerebrospinal fluid-contacting neurons-brain. Med. Acupunct. 29, 322–326. doi: 10.1089/acu.2017.1231 29067143 PMC5653342

[ref156] Wang S. Ma Z. Z. Lu Y. C. Wu J. J. Hua X. Y. Zheng M. X. . (2019). The localization research of brain plasticity changes after brachial plexus pain: Sensory regions or cognitive regions? Neural Plast. 2019:7381609. doi: 10.1155/2019/7381609 30728834 PMC6341257

[ref157] Wang Z. Nie B. Li D. Zhao Z. Han Y. Song H. . (2012). Effect of acupuncture in mild cognitive impairment and Alzheimer disease: a functional MRI study. PLoS One 7:e42730. doi: 10.1371/journal.pone.0042730 22916152 PMC3423412

[ref158] Wang L. Zhang J. Guo C. He J. Zhang S. Wang Y. . (2022). The efficacy and safety of transcutaneous auricular vagus nerve stimulation in patients with mild cognitive impairment: a double blinded randomized clinical trial. Brain Stimul. 15, 1405–1414. doi: 10.1016/j.brs.2022.09.003 36150665

[ref159] Waxenbaum J. A. Reddy V. Bordoni B. (2024). Anatomy, head and neck: cervical nerves. Treasure Island, FL: Stat pearls. 30844163

[ref160] Wen J. Chen X. Yang Y. Liu J. Li E. Liu J. . (2021). Acupuncture medical therapy and its underlying mechanisms: a systematic review. Am. J. Chin. Med. 49, 1–23. doi: 10.1142/S0192415X21500014 33371816

[ref161] White T. G. Powell K. Shah K. A. Woo H. H. Narayan R. K. Li C. (2021). Trigeminal nerve control of cerebral blood flow: a brief review. Front. Neurosci. 15:649910. doi: 10.3389/fnins.2021.649910 33927590 PMC8076561

[ref162] Wik G. Huang Y. Zeng T. Qu S. Zheng Y. Zhang J. . (2016). Waiguan stimulation may kindle Anticorrelated brain networks: functional magnetic resonance imaging data revisited. J. Acupunct. Meridian Stud. 9, 22–25. doi: 10.1016/j.jams.2015.11.035 26896073

[ref163] Wolf P. A. (2012). Contributions of the Framingham heart study to stroke and dementia epidemiologic research at 60 years. Arch. Neurol. 69, 567–571. doi: 10.1001/archneurol.2011.977 22213410 PMC3380159

[ref164] World Health Organization (1990). Who report of the working group on auricular nomenclature. Lyon: World Health Organization.

[ref165] Wu S. Y. Chen W. H. Hsieh C. L. Lin Y. W. (2014). Abundant expression and functional participation of Trpv 1 at Zusanli acupoint (St36) in mice: mechanosensitive Trpv 1 as an "acupuncture-responding channel". BMC Complement. Altern. Med. 14:96. doi: 10.1186/1472-6882-14-96 24612851 PMC3984709

[ref166] Wu X. D. Yuan J. Y. Zhao N. Q. Liu Q. G. Dong G. F. Wang X. (2021). Investigation and analysis on the current situation of clinical practice guidelines for acupuncture and moxibustion at home and abroad. Chinese Acupunct. Moxibust. 41, 923–927. doi: 10.13703/j.0255-2930.20200822-k0001 34369706

[ref167] Xia S.-H. Hu S.-W. Ge D.-G. Liu D. Wang D. Zhang S. . (2020). Chronic pain impairs memory formation via disruption of neurogenesis mediated by Mesohippocampal brain-derived neurotrophic factor signaling. Biol. Psychiatry 88, 597–610. doi: 10.1016/j.biopsych.2020.02.013 32307038

[ref168] Xu J. Wu S. Huo L. Zhang Q. Liu L. Ye Z. . (2023). Trigeminal nerve stimulation restores hippocampal dopamine deficiency to promote cognitive recovery in traumatic brain injury. Prog. Neurobiol. 227:102477. doi: 10.1016/j.pneurobio.2023.102477 37270025

[ref169] Yamada T. Yeh M. Kimura J. (2004). Fundamental principles of somatosensory evoked potentials. Phys. Med. Rehabil. Clin. N. Am. 15, 19–42. doi: 10.1016/S1047-9651(03)00100-1 15029897

[ref170] Yang F.-M. Yang Y. Daisuke W. Yi G. Xue Z. Yong-Ming G. . (2017). A summary of acupuncture standardization in Australia, Korea, Japan and the USA. World J. Acupunct. Moxibustion 27, 20–26. doi: 10.1016/S1003-5257(18)30007-2

[ref171] Yu H. Li X. Lei X. Wang J. (2019). Modulation effect of acupuncture on functional brain networks and classification of its manipulation with Eeg signals. IEEE Trans. Neural Syst. Rehabil. Eng. 27, 1973–1984. doi: 10.1109/TNSRE.2019.2939655 31502983

[ref172] Yu H. Li F. Liu J. Liu D. Guo H. Wang J. . (2024). Evaluation of acupuncture efficacy in modulating brain activity with periodic-aperiodic Eeg measurements. IEEE Trans. Neural Syst. Rehabil. Eng. 32, 2450–2459. doi: 10.1109/TNSRE.2024.3421648 38949930

[ref173] Yu H. Li F. Liu J. Liu C. Li G. Wang J. (2024). Spatiotemporal dynamics of periodic and aperiodic brain activity under peripheral nerve stimulation with acupuncture. IEEE Trans. Neural Syst. Rehabil. Eng. 32, 3993–4003. doi: 10.1109/TNSRE.2024.3492014 39499594

[ref174] Yu C. C. Ma C. Y. Wang H. Kong L. H. Zhao Y. Shen F. . (2019). Effects of acupuncture on Alzheimer’s disease: evidence from neuroimaging studies. Chin. J. Integr. Med. 25, 631–640. doi: 10.1007/s11655-018-2993-3 30155679

[ref175] Yu H. Wu X. Cai L. Deng B. Wang J. (2018). Modulation of spectral power and functional connectivity in human brain by acupuncture stimulation. IEEE Trans. Neural Syst. Rehabil. Eng. 26, 977–986. doi: 10.1109/TNSRE.2018.2828143 29752232

[ref176] Zhang X. Guo Y. Gao B. Long J. (2020). Alpha frequency intervention by electrical stimulation to improve performance in Mu-based Bci. IEEE Trans. Neural Syst. Rehabil. Eng. 28, 1262–1270. doi: 10.1109/TNSRE.2020.2987529 32305926

[ref177] Zhang Q. Sharan A. Espinosa S. A. Gallego-Perez D. Weeks J. (2019). The path toward integration of traditional and complementary medicine into health systems globally: the World Health Organization report on the implementation of the 2014-2023 strategy. J. Alternat. Complement. Med. 25, 869–871. doi: 10.1089/acm.2019.29077.jjw 31525106

[ref178] Zhang X. Su J. Gao C. Ni W. Gao X. Li Y. . (2019). Progression in vascular cognitive impairment: pathogenesis, neuroimaging evaluation, and treatment. Cell Transplant. 28, 18–25. doi: 10.1177/0963689718815820 30488737 PMC6322135

[ref179] Zhang S. Q. Wang Y. J. Zhang J. P. Chen J. Q. Wu C. X. Li Z. P. . (2015). Brain activation and inhibition after acupuncture at Taichong and Taixi: resting-state functional magnetic resonance imaging. Neural Regen. Res. 10, 292–297. doi: 10.4103/1673-5374.152385 25883630 PMC4392679

[ref180] Zheng C. X. Lu M. Guo Y. B. Zhang F. X. Liu H. Guo F. . (2016). Electroacupuncture ameliorates learning and memory and improves synaptic plasticity via activation of the Pka/Creb signaling pathway in cerebral Hypoperfusion. Evid. Based Complement. Alternat. Med. 2016:7893710. doi: 10.1155/2016/7893710, PMID: 27829866 PMC5088321

[ref181] Zheng Y. Qin Z. Tsoi B. Shen J. Zhang Z. J. (2020). Electroacupuncture on trigeminal nerve-innervated Acupoints ameliorates Poststroke cognitive impairment in rats with middle cerebral artery occlusion: involvement of neuroprotection and synaptic plasticity. Neural Plast. 2020, 1–13. doi: 10.1155/2020/8818328 PMC7492933 32963517

[ref182] Zheng W. Su Z. Liu X. Zhang H. Han Y. Song H. . (2018). Modulation of functional activity and connectivity by acupuncture in patients with Alzheimer disease as measured by resting-state fmri. PLoS One 13:e0196933. doi: 10.1371/journal.pone.0196933 29763448 PMC5953467

[ref183] Zheng Y. Wang Y. Lan Y. Qu X. Lin K. Zhang J. . (2016). Imaging of brain function based on the analysis of functional connectivity - imaging analysis of brain function by Fmri after acupuncture at Lr3 in healthy individuals. AJTCAM 13, 90–100. doi: 10.21010/ajtcam.v13i6.14 28480365 PMC5412207

[ref184] Zhong L. L. Zheng Y. Lau A. Y. Wong N. Yao L. Wu X. . (2022). Would integrated Western and traditional Chinese medicine have more benefits for stroke rehabilitation? A systematic review and meta-analysis. Stroke Vasc. Neurol. 7, 77–85. doi: 10.1136/svn-2020-000781 34446530 PMC8899656

[ref185] Zhou W. Benharash P. (2014). Effects and mechanisms of acupuncture based on the principle of meridians. J. Acupunct. Meridian Stud. 7, 190–193. doi: 10.1016/j.jams.2014.02.007 25151452

[ref186] Zhu B. Wang Y. Zhang G. Ouyang H. Zhang J. Zheng Y. . (2015). Acupuncture at Ki3 in healthy volunteers induces specific cortical functional activity: an fmri study. BMC Complement. Altern. Med. 15:361. doi: 10.1186/s12906-015-0881-3 26467429 PMC4604759

[ref187] Zlokovic B. V. Gottesman R. F. Bernstein K. E. Seshadri S. McKee A. Snyder H. . (2020). Vascular contributions to cognitive impairment and dementia (VCID): a report from the 2018 National Heart, Lung, and Blood Institute and National Institute of Neurological Disorders and Stroke workshop. Alzheimers Dement. 16, 1714–1733. doi: 10.1002/alz.12157 33030307

