# Supplementary material for: Acupuncture treatment of vascular cognitive impairment through peripheral nerve stimulation pathway: a scoping review
Source: Front Aging Neurosci. 2025 Apr 28;17:1515327. doi: 10.3389/fnagi.2025.1515327 (PMC12066784; doi:10.3389/fnagi.2025.1515327)
Supplement: Supplementary file 3 [file Data_Sheet_3.pdf]

An official website of the United States government
Here's how you know

PubMed Advanced Search Builder

PubMed®

Add terms to the query box

All Fields

Enter a search term

ADD

Show Index

Query box

Enter / edit your search query here

Add to History

History and Search Details

Search	Actions	Details	Query	Results	Time
#5	•••		Search: **((acupuncture[MeSH Terms]) OR (Acupuncture[Title/Abstract] OR Electroacupuncture[Title/Abstract] OR Acupoint[Title/Abstract] OR Acupoints[Title/Abstract] OR Electro-acupuncture[Title/Abstract] OR Eletric acupuncture[Title/Abstract])) AND (vascular cognitive impairment[Title/Abstract])**	31	09:08:31
#4	•••		Search: **vascular cognitive impairment[Title/Abstract]**	1,982	09:08:04
#1	•••		Search: **(acupuncture[MeSH Terms]) OR (Acupuncture[Title/Abstract] OR Electroacupuncture[Title/Abstract] OR Acupoint[Title/Abstract] OR Acupoints[Title/Abstract] OR Electro-acupuncture[Title/Abstract] OR Eletric acupuncture[Title/Abstract])**	41,394	08:30:28

Showing 1 to 3 of 3 entries
